# Positively Charged Additives Facilitate Incorporation in Inorganic Single Crystals

**DOI:** 10.1021/acs.chemmater.2c00097

**Published:** 2022-05-18

**Authors:** Ouassef Nahi, Alexander Broad, Alexander N. Kulak, Helen M. Freeman, Shuheng Zhang, Thomas D. Turner, Lucien Roach, Robert Darkins, Ian J. Ford, Fiona C. Meldrum

**Affiliations:** † School of Chemistry, University of Leeds, Woodhouse Lane, Leeds LS2 9JT, U.K.; ‡ Department of Physics and Astronomy, University College London, Gower Street, London WC1E 6BT, U.K.; § School of Civil Engineering, University of Leeds, Woodhouse Lane, Leeds LS2 9JT, U.K.; ∥ Université de Bordeaux, CNRS, Bordeaux INP, ICMCB, UMR 5026, 33600 Pessac, France

## Abstract

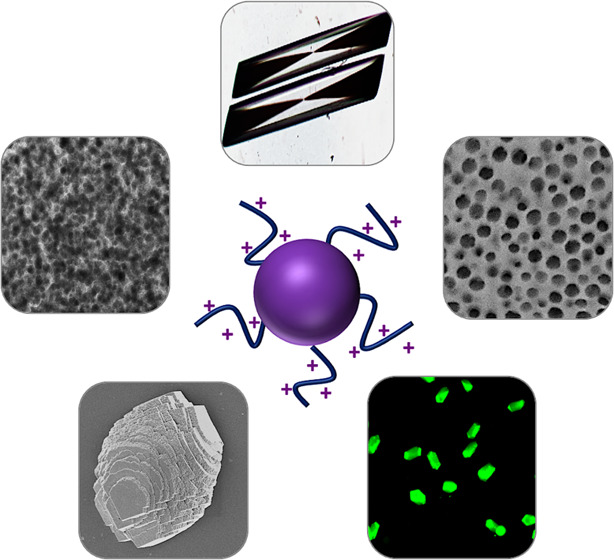

Incorporation of guest additives within inorganic single crystals offers a unique strategy for creating nanocomposites with tailored properties. While anionic additives have been widely used to control the properties of crystals, their effective incorporation remains a key challenge. Here, we show that cationic additives are an excellent alternative for the synthesis of nanocomposites, where they are shown to deliver exceptional levels of incorporation of up to 70 wt % of positively charged amino acids, polymer particles, gold nanoparticles, and silver nanoclusters within inorganic single crystals. This high additive loading endows the nanocomposites with new functional properties, including plasmon coupling, bright fluorescence, and surface-enhanced Raman scattering (SERS). Cationic additives are also shown to outperform their acidic counterparts, where they are highly active in a wider range of crystal systems, owing to their outstanding colloidal stability in the crystallization media and strong affinity for the crystal surfaces. This work demonstrates that although often overlooked, cationic additives can make valuable crystallization additives to create composite materials with tailored composition–structure–property relationships. This versatile and straightforward approach advances the field of single-crystal composites and provides exciting prospects for the design and fabrication of new hybrid materials with tunable functional properties.

## Introduction

Incorporation of additives within single crystals holds enormous potential for the design and fabrication of composite materials with superior optical, electrical, mechanical, and catalytic properties.^1−7^ The single-crystal matrix ensures that the additives are completely isolated from the surrounding environment, and the hybrid crystals possess structures that could not be replicated by simple mixing of the individual components. Incorporation of amino acids in calcite (CaCO_3_) enhances the hardness of the hybrid crystals,^2^ while improved stability and increase in band gap are achieved for organometal halide perovskites and ZnO crystals.^8−10^ The occlusion of functional nanoparticles is particularly attractive, as their properties can be predefined using well-established synthetic methods. For example, the incorporation of metal oxide nanoparticles^11−13^ in KH_2_PO_4_ and K_2_SO_4_ has created single crystals for optoelectronic and photonic devices, while calcite-containing polymer nano-objects^14^ and magnetic nanoparticles^15^ exhibit enhanced physical properties. Incorporation of gold nanoparticles^3^ in ZnO crystals delivered nanocomposites with tunable band gaps and superior photocatalytic activity.

Despite these achievements, the occlusion of high concentrations of additives in single crystals remains challenging.^16−18^ Indeed, only a few weight percent of quantum dots, Au, and Fe_3_O_4_ nanoparticles were incorporated within calcite,^19,20^ Cu_2_O,^21,22^ and NaCl and borax^7^ single crystals using gel-trapping, confinement-based, and evaporation strategies, respectively. Nevertheless, it is possible to achieve homogeneous incorporation using simple coprecipitation,^3,23−25^ provided that an appropriate balance is achieved between additive binding and the rate of crystal growth. The additives must bind sufficiently strongly to the crystal surface such that they are engulfed by the subsequent steps but not so strongly that they inhibit crystal growth.^26−28^

This strategy has been elegantly demonstrated for CaCO_3_, where a broad range of organic (e.g., polymer nanoparticles and protein nanogels)^14,23,29,30^ and inorganic (e.g., carbon dots, Au, and Fe_3_O_4_) nanoparticles^4,15,24^ have been incorporated within calcite single crystals. Occlusion was achieved by functionalizing the nanoparticles with anionic polyelectrolytes rich in carboxylate,^30−33^ sulfate,^34^ sulfonate,^35^ or phosphate^36^ functional groups such that they bind strongly to the crystal surfaces. However, this could only be carried out at low solution supersaturations and particle concentrations due to the colloidal instability of these anionic particles in the presence of calcium ions.^26^ This, in turn, limits the scalability and thus the practical applications of this approach. Nanoparticles are therefore required that have a high affinity for the crystal surface and that retain their colloidal stability at high calcium ion concentrations.^24^

Here, we show that these challenges can be overcome by functionalizing the particulate additives with cationic polyelectrolytes. Exceptional levels of incorporation (up to 70 wt %) of latex particles, gold nanoparticles, and silver nanoclusters are achieved within calcite and alternative carbonate, sulfate, and oxide single crystals. Insight into the mechanism of occlusion and the structure/composition relationships of the nanocomposites is obtained from molecular dynamics simulations and synchrotron high-resolution powder X-ray diffraction. We also demonstrate that such high nanoparticle loadings lead to nanocomposites with new properties, including bright fluorescence and surface-enhanced Raman scattering (SERS). While anionic molecules have been the predominant choice of crystallization additive for inorganic compounds, this work shows that cationic species can outperform their anionic counterparts in some applications.

## Results

### Incorporation of Small Basic Molecules in Calcite

**Figure 1:**
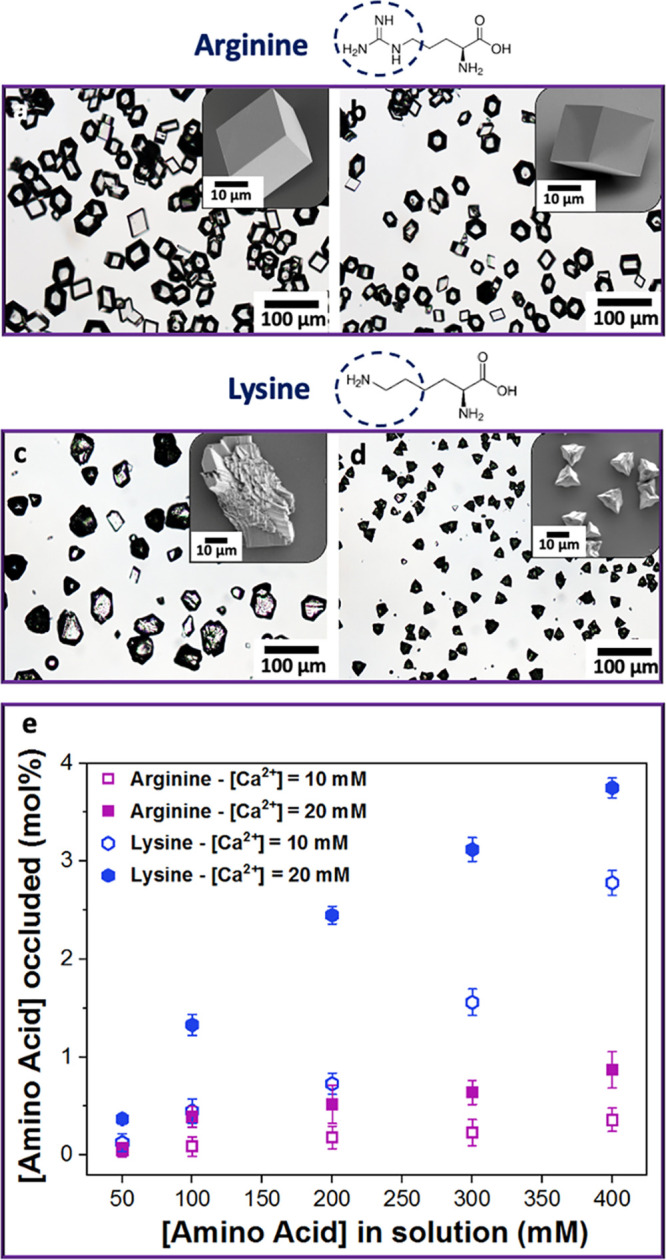
Optical micrographs of calcite precipitated in the presence of (a, b) [l-arginine] = 400 mM, (c, d) [l-lysine] = 400 mM and (a, c) [Ca^2+^] = 10 mM and (b, d) [Ca^2+^] = 20 mM. Insets are the SEM images of the calcite crystals. (e) Graph showing the relationship between occlusion and the concentration of amino acid in solution.

Initial experiments investigated the precipitation of calcium carbonate in the presence of the basic amino acids l-arginine (Arg) and l-lysine (Lys). Precipitation was carried out using the ammonia diffusion method (pH ≈9),^37^ where this yielded calcite under all of the conditions investigated ([Ca^2+^] = 10–20 mM and [amino acid] = 50–400 mM) (Figures 1, S1, and S2). Arg had no effect on the crystal morphologies, while Lys yielded rough crystals that were highly elongated along their *c*-axes. This morphology change is indicative of the preferential interaction of Lys with the acute over the obtuse steps of calcite.^26,38^ Thermogravimetric analyses (TGA) of crystals grown at [Ca^2+^] = 20 mM and [amino acid] = 400 mM revealed that only ≈0.8 mol % of Arg incorporated in calcite, while up to ≈4 mol % was recorded for Lys (Figures 1e and S3).

**Figure 2:**
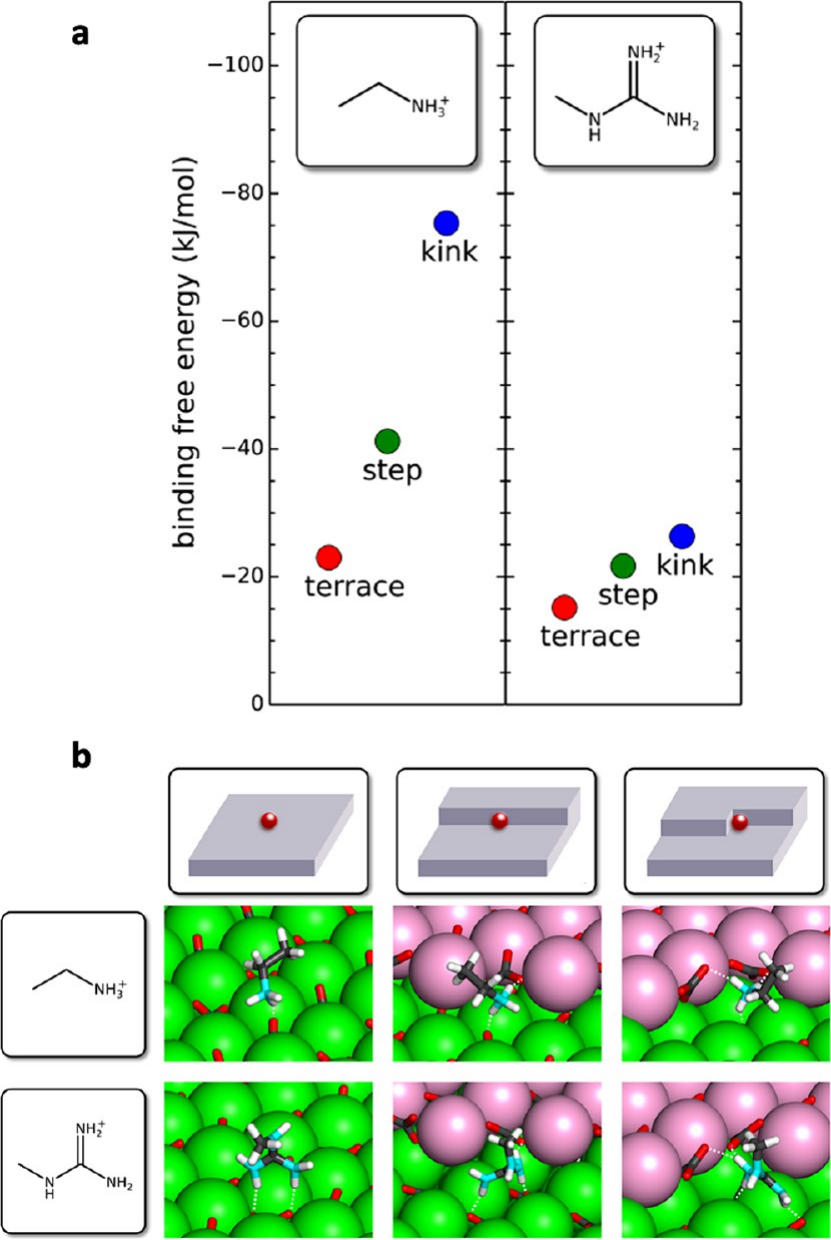
(a) Binding free energies for molecules representing the sides chains of l-lysine and l-arginine with the carbonate anions at the calcite terrace, acute step, and acute kink adsorption sites. (b) Snapshots from trajectories showing binding to the calcite terrace, step, and kink sites. The white dashed lines indicate hydrogen bonds. Calcium is shown in green for the lower terrace and pink for the upper terrace. Carbon is shown in gray, oxygen in red, nitrogen in cyan, and hydrogen in white. Water is excluded from all images for clarity.

Molecular simulations were performed to elucidate the contrasting effects of Lys and Arg. The binding free energies for molecules representing the Lys and Arg side chains were computed at the terrace, acute step, and acute kink sites of calcite (Figure 2a). Both molecules were modeled in a protonated state (p*K*_a_ side chain of Lys = 10.6 and Arg = 12), reflecting the pH of the experiments. The primary (−NH_3_) amine in the Lys side chain interacts strongly with the carbonate anions at all adsorption sites; the adsorption free energy increased roughly in proportion to the number of carbonate anions available—one for a terrace, two for a step, and three for a kink (Figure 2b). The calcite surface is also expected to be negatively charged at the experimental pH of ≈9,^37^ which will further promote the association of Lys with the calcite surface. In contrast, the binding free energy of the Arg side chain was weaker at all adsorption sites, especially the step and kink sites. The stereochemistry and charge density of the single amine in the Lys side chain therefore provides stronger binding to calcite than the three amines in the Arg side chain, which is consistent with its enhanced occlusion and morphological effects.

### Incorporation of Cationic Polymer Nanoparticles in Calcite

**Figure 3:**
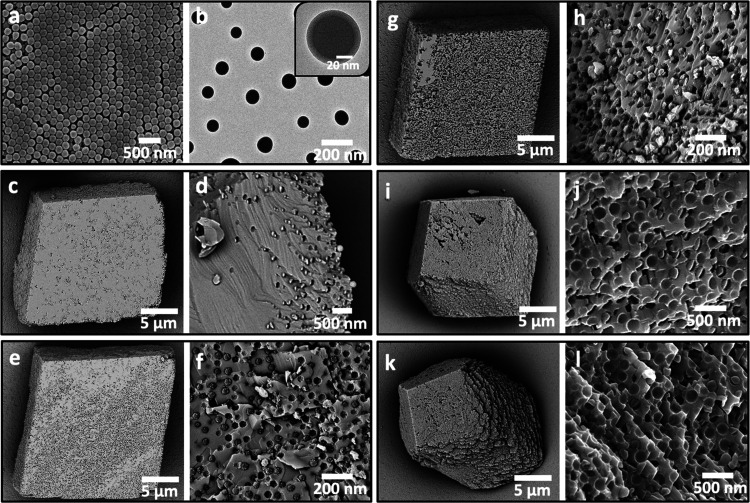
(a) SEM micrograph of the cationic PMMA-PEI nanoparticles. (b) TEM image showing that the nanoparticles are core–shell structures with a 15 nm PEI corona. (c–l) SEM images and cross sections through PMMA-PEI/calcite composites precipitated in the presence of (c–h) 0.10 wt % and (i–l) 0.25 wt % PMMA-PEI nanoparticles for (c, d) [Ca^2+^] = 1.5 mM, (e, f, i, j) [Ca^2+^] = 2.5 mM, and (g, h, k, l) [Ca^2+^] = 5 mM.

The incorporation of larger cationic particulate additives was then assessed. Poly(methyl methacrylate) (PMMA) nanoparticles that were surface-functionalized with branched poly(ethyleneimine) (PEI) were synthesized.^39^ Scanning electron microscopy (SEM) and transmission electron microscopy (TEM) showed that the dry particles were highly monodisperse 150 nm spheres (Figure 3a,b) with a well-defined core/shell structure and a ≈15 nm PEI coating (Figure 3b). Dynamic light scattering (DLS) of the particles in water confirmed their uniformity (polydispersity index (PDI) = 0.05) and a mean hydrodynamic diameter of 200 nm (Figure S4), while ζ-potential measurements showed that they were positively charged at the pH = 9 used for CaCO_3_ precipitation (+45.7 mV).^40^

Calcite crystals precipitated in the presence of the nanoparticles were elongated along the *c*-axes and capped by {104} faces (Figure 3). Greater morphological effects were achieved at higher nanoparticle concentrations and supersaturations (Figure 3c–l) as the latter increases the step density, and therefore the growth rate, of the {104} faces, reducing their expression in the morphology. This morphological change is again indicative of preferential binding of the particles to the acute over the obtuse steps of calcite, as has been observed in *in situ* AFM studies of calcite growth in the presence of nanoparticles. Binding is through the PEI chains on the surfaces of the particles,^2,24,41−43^ where the specificity is expected to be sensitive to the reaction conditions.^44^ As indicated by the macroscopic roughening of the crystal surfaces, these additives must induce the formation of macro-steps and, ultimately, step-bunching, where there may be greater specificity and stronger binding to the larger macro-steps.

DLS analyses revealed that the cationic nanoparticles remained highly stable in solutions containing calcium and carbonate ions at concentrations as high as 50 mM (Figures S6 and S7). Such excellent colloidal stability in the mineralization solution enables their effective binding and entrapment within calcite, as shown by examining cross sections through the crystals, prepared by focused ion beam (FIB) milling or fracturing. The particles were uniformly incorporated (Figure 3c–j). TGA further confirmed high particle loadings in the calcite crystals, where they comprise up to 33 wt % (57 vol %) cationic nanoparticles (Figure S5).

### Comparison with Low Charge and Anionic Polymer Nanoparticles

**Figure 4:**
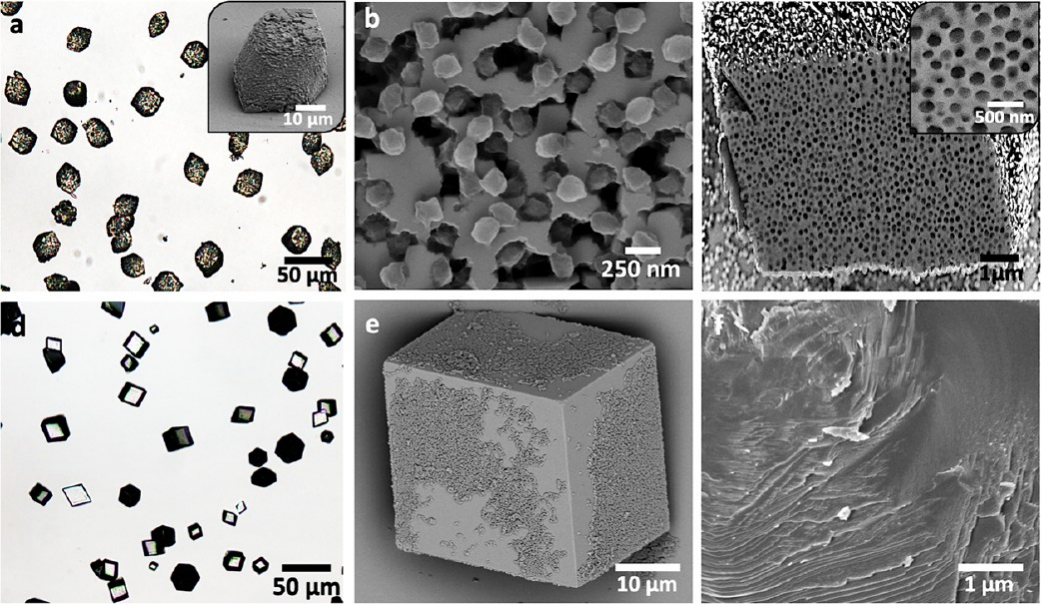
Optical (a, d) and SEM micrographs (inset in a and b, c, e, f) of calcite crystals precipitated in the presence of (a–c) PMMA-PEI nanoparticles and (d–f) nonfunctionalized PS nanoparticles at [Ca^2+^] = 10 mM and [nanoparticles] = 0.5 wt %. (b) Surface of PMMA-PEI/calcite nanocomposites showing embedded nanoparticles. (c) Cross section showing uniform incorporation of the PMMA-PEI nanoparticles in calcite. (d, e) Images of unmodified calcite crystals precipitated in the presence of nonfunctionalized PS nanoparticles and (f) cross section showing no incorporation of nanoparticles in the bulk of the calcite crystals.

**Figure 5:**
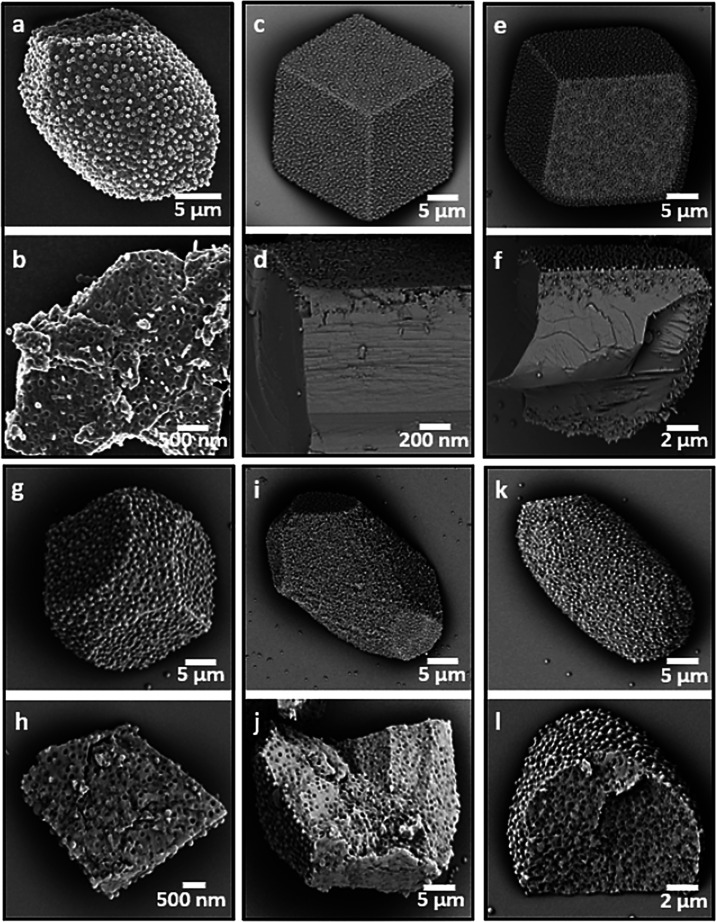
SEM micrographs of calcite crystals precipitated at [Ca^2+^] = 1.5 mM (a, b) and [Ca^2+^] = 2.5 mM (c–l) in the presence of carboxyl-functionalized nanoparticles at concentration (a–d) 0.1 wt %, (e, f) 0.25 wt %, (g, h) 0.5 wt %, and (i, j) 1 wt %, (k, l) 2.5 wt %. SEM of fractured crystals showing that efficient incorporation is achieved at [Ca^2+^] = 1.5 mM and [nanoparticles] = 0.1 wt % (b), whereas only surface-confined occlusion is achieved at [Ca^2+^] = 2.5 mM and [nanoparticles] ≤ 0.25 wt % (d and f). Uniform incorporation occurs at [Ca^2+^] = 2.5 mM and [nanoparticle] ≥ 0.5 wt %.

The incorporation of the cationic PMMA-PEI nanoparticles (Figure 4a–c) was then compared with 200 nm low-charge and anionic nanoparticles. Nonfunctionalized poly(styrene) nanoparticles failed to incorporate or alter the crystal morphology (Figures 4d–f and S5), while carboxyl-functionalized nanoparticles induced the same pattern of morphological changes as the cationic nanoparticles (Figure 5). Although the anionic particles incorporated better than cationic particles at low calcium ion concentrations ([Ca^2+^] = 1.5 mM and [particles] = 0.1 wt %), a small increase to [Ca^2+^] = 2.5 mM resulted in a reversal of their activities. Homogeneous incorporation of cationic particles occurred at [Ca^2+^] = 2.5 mM and [particles] = 0.1 wt %, while 0.5 wt % of the anionic particles was required to give equivalent occlusion under the same conditions. This is attributed to the excellent colloidal stability of the cationic particles in the crystallization solution, which ensures that they remain well-dispersed in solutions of [Ca^2+^] ≤ 50 mM (Figure S6). In contrast, the carboxyl-functionalized nanoparticles aggregated at a much lower concentration of [Ca^2+^] > 5 mM (Figure S8). Strong complexation of the carboxylated nanoparticles with Ca^2+^ ions in solution also screens the negative charges and hampers their binding to the crystal surface at higher calcium concentrations.

### Incorporation of Cationic Metal Nanoparticles in Calcite

**Figure 6:**
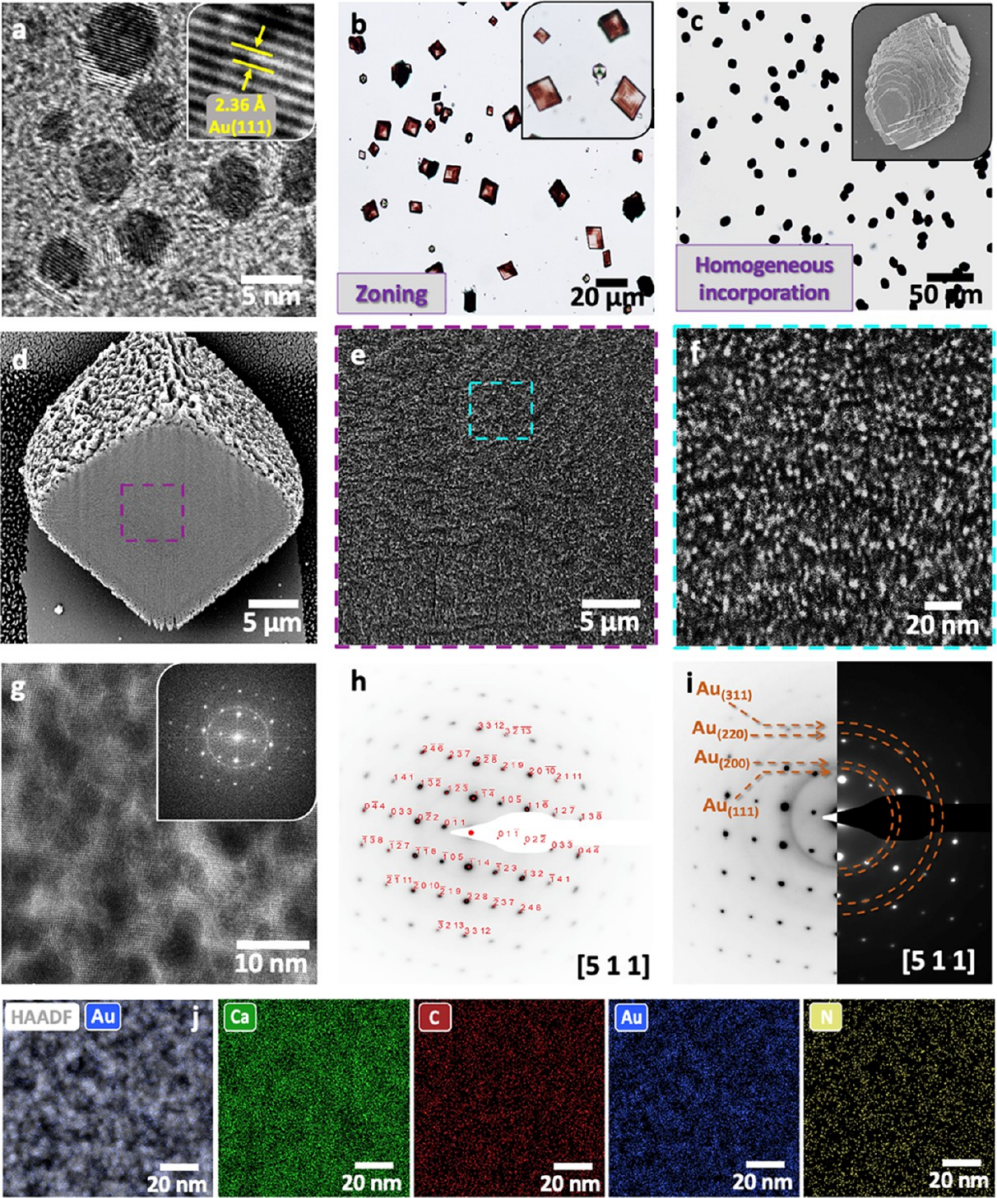
(a) TEM image of ≈5 nm AuNPs functionalized with branched PEI (Au/PEI). The inset is a HRTEM image showing Au (111) lattice fringes. (b) Optical image of calcite crystals precipitated at [Ca^2+^] = 10 mM and [Au/PEI] = 0.01 wt %, showing zoning effects. (c) Optical micrograph and SEM image (inset) of dark and elongated calcite crystals precipitated at [Ca^2+^] = 10 mM and [Au/PEI] = 0.1 wt %. (d–f) FIB-SEM images through crystals revealing the dense and uniform incorporation of the AuNPs (bright spots) in calcite. (g) HRTEM and corresponding FFT of a cross section through a nanocomposite crystal, displaying lattice fringes that demonstrate the single crystallinity of the calcite host. (h, i) SAED patterns of the nanocomposites showing diffraction spots of calcite single crystals and rings corresponding to Au. (j) HAADF-STEM and EDX-STEM maps showing the uniform distribution of Ca, C (i.e., calcite) and Au, N (i.e., Au/PEI nanoparticles) throughout the hybrid crystals.

This approach was then extended to create functional nanocomposite crystals using cationic metal nanoparticles.^24,45,46^ Gold nanoparticles (AuNPs) functionalized with branched PEI (Au/PEI) were synthesized by complexing hydrochloroauric acid with branched PEI (*M*_W_ = 1200 g mol^–1^) and then reduced with sodium borohydride. The average AuNPs diameter was 5 nm (±1 nm), as determined by TEM (Figure 6a). The AuNPs were highly stable in the mineralization solution, where DLS revealed only a minor increase in the hydrodynamic diameter from ≈6.5 to 8 nm as [Ca^2+^] was increased from 0 to 30 mM (Figure S9). This is due to the slight expansion of the PEI polymers capping the AuNPs. ζ-potential analysis confirmed that the nanoparticles were positively charged at pH = 9 (Figure S8), and the invariance of the surface plasmon resonance band at *λ* = 520 nm demonstrated their stability in aqueous solutions containing [Ca^2+^] and [CO_3_^2–^] ≤ 50 mM (Figures S10 and S11). This is a significant advantage over occlusion systems based on nanoparticles functionalized with anionic polymers, where these readily aggregate in the presence of metal ions.^24,35^

Pink calcite crystals with nonuniform colors characteristic of intra-sectoral zoning formed on coprecipitation at very low [Au/PEI] = 0.01 wt % (Figure 6b), while higher [Au/PEI] = 0.1 wt % yielded elongated, dark red/black crystals (Figure 6c). The AuNPs were uniformly distributed throughout the host crystal (Figures 6d–f,j and S12), and the analysis of bulk samples using atomic absorption spectroscopy (AAS) and TGA showed that they comprise ≈43 wt % Au and ≈28 wt % PEI (Figure S13), and thus a remarkable 70 wt % of foreign material in calcite. While one may intuitively expect a disruption of the structure of the host crystal, both high-resolution TEM (HRTEM) (Figure 6g) and selected area electron diffraction (SAED) (Figure 6h,i) of a thin section cut from a nanocomposite crystal demonstrates that the single-crystal structure of the calcite host is preserved.

**Figure 7:**
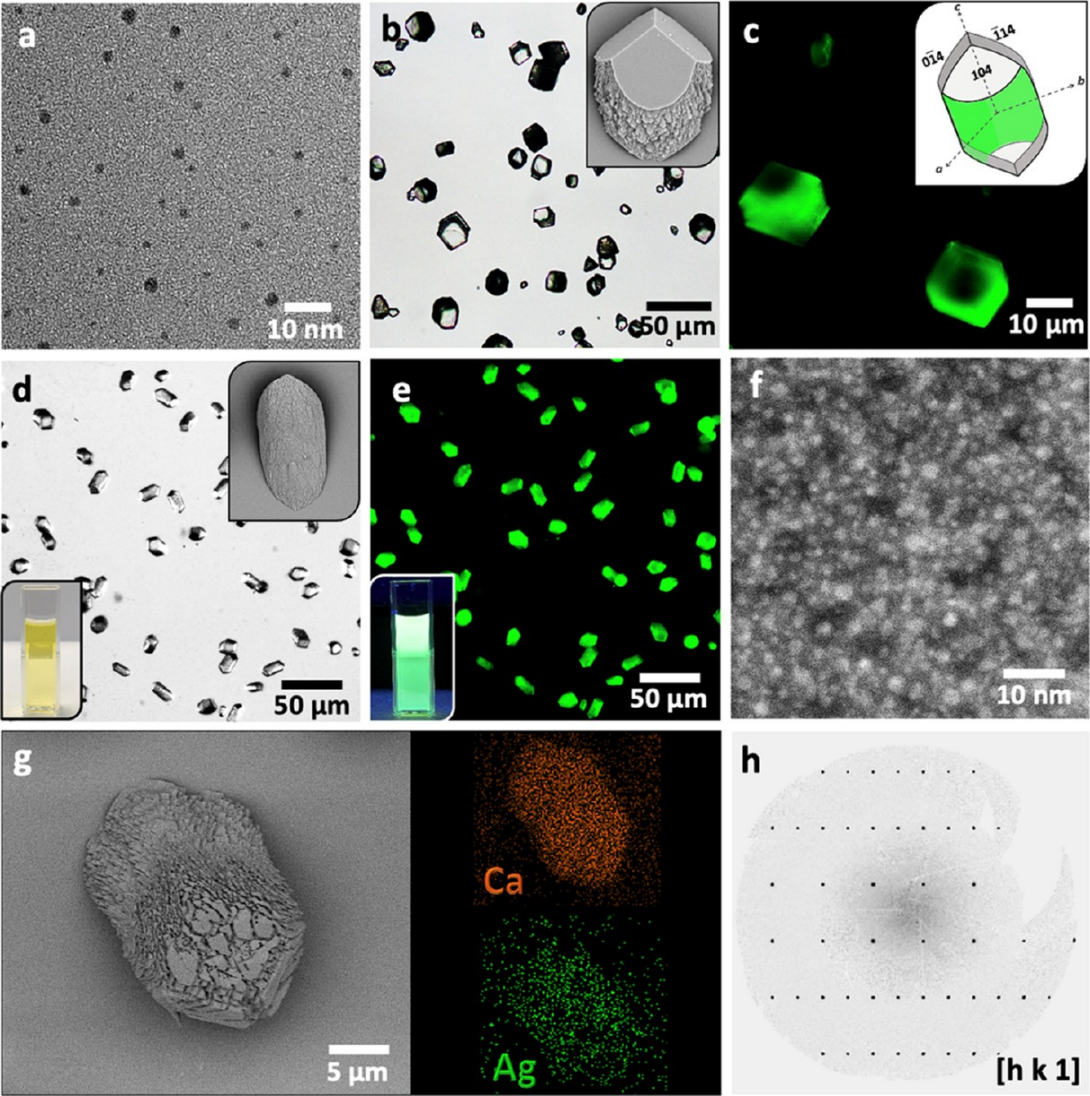
(a) TEM micrograph of Ag nanoclusters functionalized with branched PEI (Ag/PEI). (b) Optical and SEM (inset) images of calcite precipitated at [Ca^2+^] = 10 mM and [Ag/PEI] = 0.01 wt % and (c) corresponding fluorescence image of the hybrid crystals showing zoning. (d) Optical image, SEM image (top right), and digital image (bottom left) of calcite precipitated at [Ca^2+^] = 10 mM and [Ag/PEI] = 0.1 wt %, and corresponding (e) fluorescence and (f) dark-field TEM images demonstrating homogeneous incorporation of Ag/PEI. (g) SEM-EDX maps showing elemental Ca and Ag of the composite crystals precipitated at [Ca^2+^] = 10 mM and [Ag/PEI] = 0.1 wt %, and (h) corresponding XRD pattern demonstrating the single-crystal character of the hybrid crystals.

This approach was also successfully applied to yet smaller silver nanoparticles (Ag/PEI) prepared by slow reduction of a solution of silver nitrate and branched PEI (*M*_W_ = 10 000 g mol^–1^) with l-ascorbic acid. This gave Ag nanoclusters with <2 nm Ag cores (Figures 7a and S15) and ≈4 nm hydrodynamic diameter (Figure S14), which exhibit bright fluorescence under UV irradiation (*λ* = 365 nm) (inset Figure 7d).^47^ Comparison of calcite growth in the presence of [nanoparticles] = 0.01 wt % showed that the Ag/PEI and Au/PEI nanoparticles induced the same pattern of morphological changes (Figure 7b) but that the Ag/PEI clusters were more effective. This is attributed to a higher number density of the smaller Ag/PEI nanoclusters. These crystals again exhibited zoning, where the nanoclusters concentrated in the rough equatorial region of calcite (Figure 7c).

Higher [Ag/PEI] = 0.1 wt % resulted in their uniform incorporation (Figures 7d–g and S16) and endowed the host crystal with bright fluorescence under UV light (Figure 7e). Analysis of bulk samples using inductively coupled plasma-optical emission spectroscopy (ICP-OES) revealed Ag incorporation levels of 37.5 wt %, and TGA showed that the crystals comprised 26.3 wt % PEI (Figure S13). This again demonstrates that exceptional amounts—64 wt %—of Ag clusters can be incorporated into calcite while preserving the single-crystal structure (Figure 7h).

### Microstructure of the Nanocomposite Crystals

**Figure 8:**
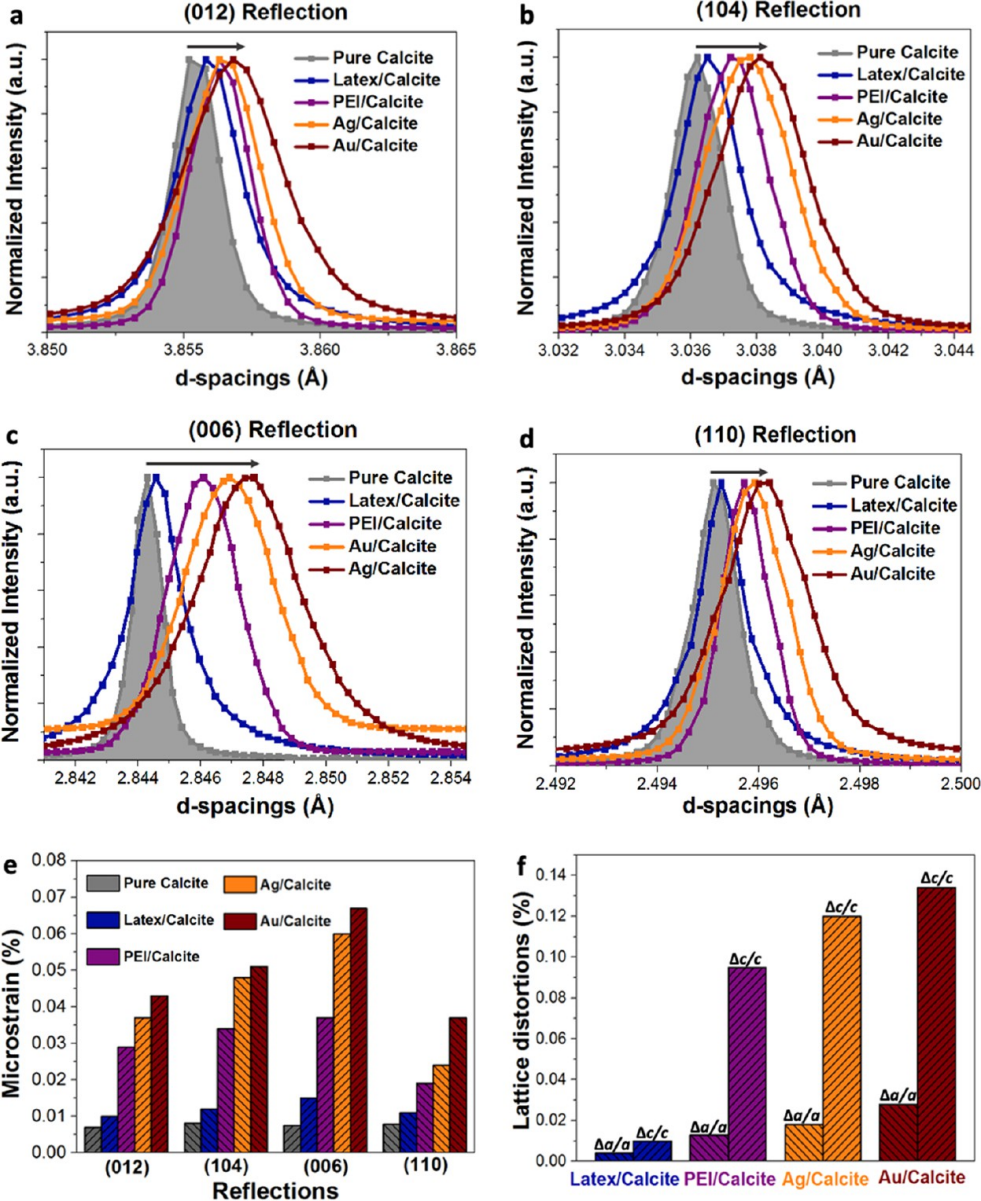
(a–d) HRPXRD patterns and (e) microstrain fluctuations of the indicated reflections of pure calcite (gray), PMMA-PEI latex/calcite (blue), PEI/calcite (purple), Ag/calcite (orange), and Au/calcite (red). (f) Lattice distortions along the *a*-axis and *c*-axis of calcite incorporating the diverse cationic additives.

The influence of the occluded PMMA-PEI, Au/PEI, and Ag/PEI nanoparticles on the structure of the calcite host crystal was investigated using synchrotron high-resolution powder X-ray diffraction (HRPXRD), where data were compared with pure calcite and calcite containing PEI (Figures 8 and S18). Lattice distortions, microstrain fluctuations, and coherence lengths were measured for the (012), (104), (006), and (110) reflections, giving information about the additive/crystal interactions along different crystallographic directions.

PMMA-PEI nanoparticles only had minor effects on the calcite structure even at incorporation levels of 57 vol %, causing a decrease in coherence lengths from 650 nm in pure calcite to 540 nm and small lattice distortions of Δ*a*/*a* = 0.004% and Δ*c*/*c* = 0.01%. This is consistent with previous analysis of polymer nano-objects/calcite composites.^30^ By comparison, the Bragg peaks of calcite containing ≈18 wt % PEI were significantly broadened and shifted to larger *d*-spacings. This sample also showed smaller coherence lengths of 470 and 330 nm perpendicular to the (110) and (006) planes, respectively, and lattice expansions of Δ*a*/*a* = 0.013% and Δ*c*/*c* = 0.095%. The greater lattice distortions along the *c*-axis than the *a*-axis (Figure 7f) are consistent with the elastic anisotropy of calcite.^2^

Incorporation of 64 wt % Ag/PEI and 70 wt % Au/PEI nanoparticles yielded significant broadenings and shifts of the diffraction peaks toward larger d-spacings. Coherence lengths were ≈350 and ≈210 nm perpendicular to the (110) and (006) planes for both nanocomposites, and lattice expansions reaching up to Δ*c*/*c* = 0.135% and Δ*a*/*a* = 0.03% were recorded. These values are high for a brittle ceramic and are comparable to those measured in calcite biominerals occluding biomolecules.^48^ The strong influence of these small particles can be attributed to the large additive/host interfacial area, where the specific surface areas of the ≈6.5 nm Au/PEI particles and ≈4 nm Ag/PEI are ≈1000 times and ≈2500 times larger than that of the 200 nm latex particles, respectively. That the Au/PEI nanoparticles induced slightly greater lattice expansions and microstrains may derive from the composition of the nanoparticles, where the Ag/PEI nanoclusters comprise a higher percentage of polymer than the Au/PEI nanoparticles. This suggests that the metal nanoparticle core and the polymer coating have different effects on the crystal lattice.

### Incorporation in Alternative Host Crystals

**Figure 9:**
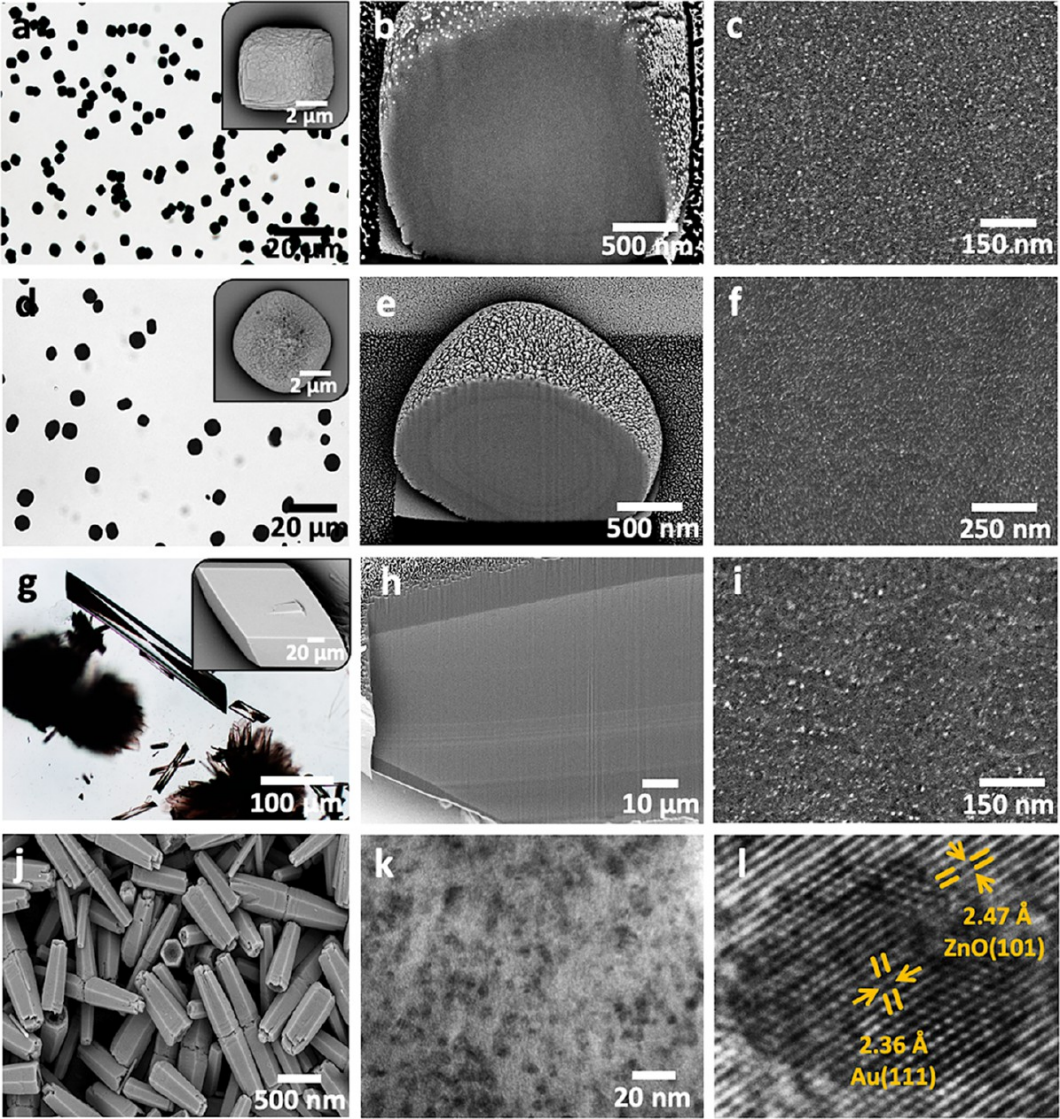
(a–c) MnCO_3_, (d–f) SrSO_4_, (g–i) CaSO_4_·2H_2_O and (j–l) ZnO nanocomposite crystals incorporating Au/PEI nanoparticles. (a, d, g) Optical images (insets are SEM images), (j) a SEM image of the Au/ZnO nanocrystals. (b, c, e, f, h, i) FIB-SEM cross-section images revealing the high levels of AuNP incorporation (bright spots) in the various inorganic host crystals. (k) TEM micrograph of a thin section through an Au/ZnO nanocomposite showing the uniformly incorporated nanoparticles (l) and corresponding HRTEM image displaying the lattice fringes of Au (111) and ZnO (101).

The versatility of this approach was then demonstrated by extending it to manganese carbonate (MnCO_3_, rhodochrosite), strontium sulfate (SrSO_4_, celestine), calcium sulfate dihydrate (gypsum), and zinc oxide (ZnO, zincite) (Figures 9 and S19–S22). Incorporation of AuNPs within MnCO_3_, SrSO_4_, and CaSO_4_·2H_2_O was achieved by mixing 0.1 wt % Au/PEI with the respective crystallization solutions. Au/ZnO nanocomposites were prepared under mild hydrothermal conditions in which 1 wt % Au/PEI was added to an aqueous solution of zinc nitrate hexahydrate and HMTA, and the reaction mixture was heated at 90 °C for 90 min. High levels of occlusion were achieved in all crystals. The nanoparticles were uniformly distributed throughout ZnO, MnCO_3_, and SrSO_4_, where the ZnO contained ≈22.5 wt % Au/PEI, and the MnCO_3_ and SrSO_4_ over ≈65 wt %. Gypsum displayed pronounced sectoral zoning (Figure 9g) that is common for this crystal and derives from preferential binding of the AuNPs to the (011) planes.^49^ Increasing the amount of Au/PEI in solution to 0.25 wt % enabled uniform incorporation of the AuNPs in gypsum (≈47 wt %) (Figure S22).

### Optical Properties of the Nanocomposite Crystals

**Figure 10:**
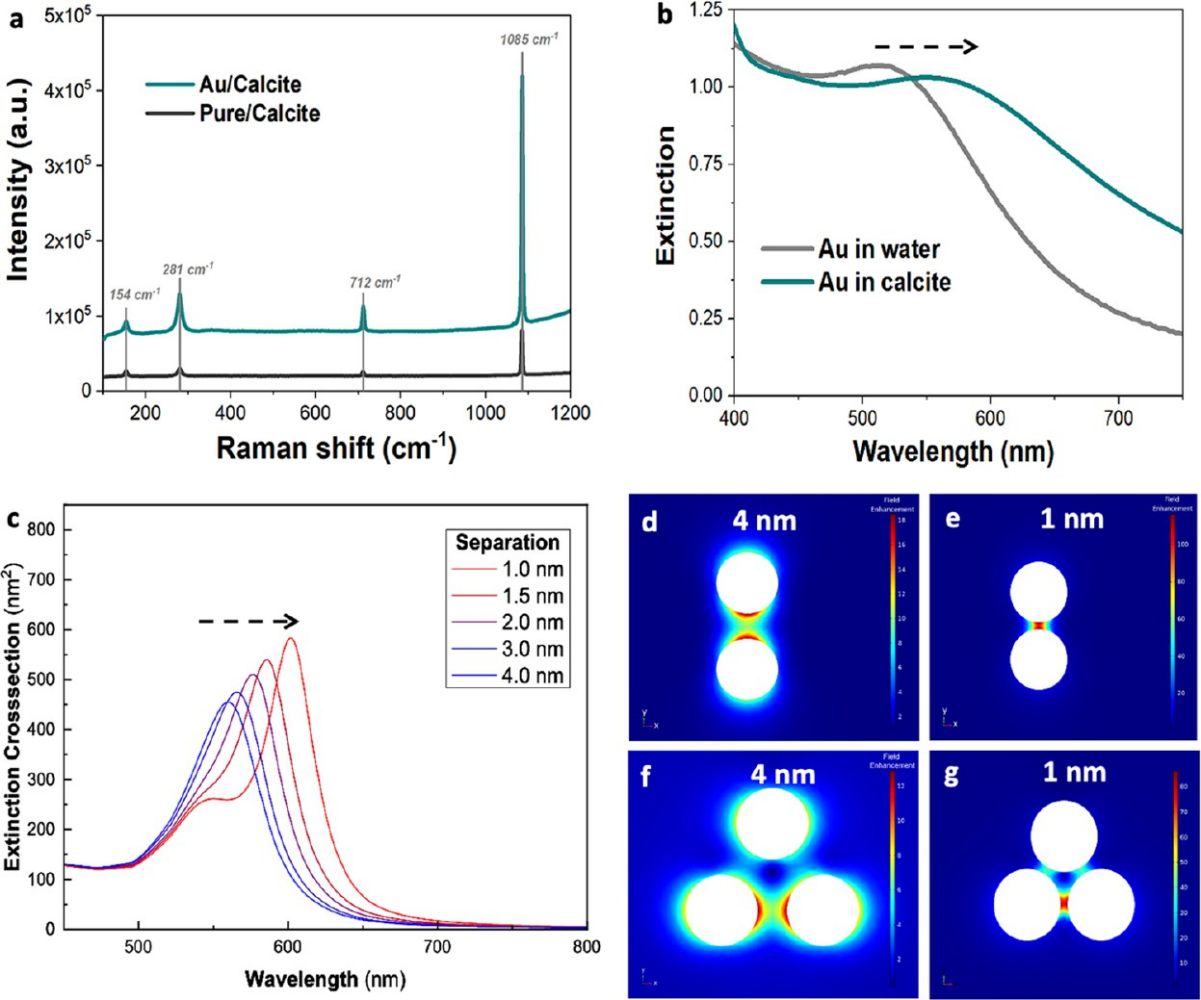
(a) Raman spectra of pure crystals (gray) and Au/calcite nanocomposites (cyan), showing a surface-enhanced Raman scattering (SERS) effect for the composites when irradiated with a laser source (λ = 785 nm) using 10 s exposure time and 5% laser power. The spectra are offset for clarity. (b) UV–visible extinction spectra of Au/PEI in pure water (gray) and in calcite (cyan), displaying a red shift of the surface plasmon resonance band of the AuNPs when incorporated in calcite. (c) Simulated extinction spectra for 5 nm AuNPs dimers occluded in calcite with varying the interparticle separation between 1 and 4 nm. (d–g) FEM-simulated field enhancement |*E*|/|*E*_0_| along the *y*-axis for the 5 nm AuNPs dimers (d, e) and trimers (f, g) with an interparticle gap of 4 nm (d, f) and 1 nm (e, g).

The experimental extinction spectra of the Au/calcite crystals show that the Au plasmon peak is broadened and shifted to ≈557 nm as compared with ≈520 nm for AuNPs dispersed in an aqueous solution (Figure 10b). This is consistent with finite element simulations of a pair of 5 nm AuNPs embedded in calcite, which predict a red shift in the resonance peak from 520 to 560 nm due to the high refractive index of the calcite (*n* = 1.66) when the AuNPs are separated by 4 nm, and ultimately, the development of two separate peaks as the interparticle distance is decreased (Figure 10c).

The nanocomposite crystals also display surface-enhanced Raman scattering (SERS),^50^ where this effect arises from the close proximity of the AuNPs (down to ≈1 nm) in the host crystals (Figure 10a), creating intense localized electromagnetic fields in the regions between the AuNPs.^50,51^ The main Raman peaks (i.e., 1085 cm^–1^ for calcite and MnCO_3_, 1002 cm^–1^ for SrSO_4_, 1008 cm^–1^ for gypsum, and 437 cm^–1^ for ZnO) of the composites are significantly more intense than those of the pure crystals, with enhancement factors of 2 and 4.5 for ZnO and gypsum, respectively, and ≈6 for calcite, MnCO_3_, and SrSO_4_ (Figures 10a and S19–S22). An inverse relationship exists between the SERS signal enhancement and the interparticle distance between adjacent AuNPs such that the values recorded scale with the levels of Au incorporation within the various host crystals.^50,52^

The experimental SERS data were rationalized by the simulations, where the increase in the Raman signals, ^em^*G*_SERS_, is proportional to the fourth power of the local field enhancement, |*E*|/|*E*_0_|, where *E* is the local electric field and *E*_0_ is the incident electric field.^53^ The simulations predict field enhancements ^em^*G*_SERS_ ≈ 10^3^ at the midpoint between two AuNPs separated by 4 nm, where the induced field is inversely proportional to the third power of the distance from the particle surface.^54^ Hot spots on the AuNPs surfaces with ^em^*G*_SERS_ of 10^5^–10^8^ are also predicted for particle separations of 1–4 nm (Figure 10d–g), but these are unlikely to be fully accessible by the calcite crystal due to the PEI coating on the AuNPs. The field enhancement observed in the nanocomposite will also be reduced due to the presence of multiple AuNPs in close proximity, which will dilute the peak field enhancement and lower ^em^*G*_SERS_ (Figures S23 and S24).

## Discussion

Incorporation of soluble additives within inorganic single crystals holds enormous potential for the synthesis of hybrid materials with advanced properties. However, the creation of such materials is far from trivial, as the simple addition of particles to a crystallization solution typically leads to low levels of occlusion.^16,17^ This has been addressed by functionalizing the particles with block copolymers that modify the interaction of the particles with the crystal surface to enhance their occlusion and ensure their colloidal stability in the crystallization solution.^26,35^

Anionic polyelectrolytes have been used extensively to functionalize the particles and drive incorporation.^18,32−36^ This selection is made based on the traditional choice of anionic additives for controlling CaCO_3_ precipitation, where they are often highly active in modifying morphologies and retarding nucleation.^26,35^ Highly acidic macromolecules are also characteristic of calcium carbonate biomineralization and are thus often employed in bio-inspired mineralization.^14,23,30^ However, solution conditions are restricted to low supersaturations and particle concentrations as strong electrostatic interactions between the calcium ions and anionic moieties on the surface of the particles cause particle aggregation. This significantly limits the particle loading and yield of these nanocomposites.

Many studies have been performed to determine “design rules” that govern the occlusion of nanoparticles functionalized with polymer chains. As a general rule, short anionic polymer chains often lead to occlusion in the outer surface of the calcite crystals only,^17,26,35^ while longer chains deliver incorporation throughout the crystals.^14,26,30^ This behavior has been attributed to the higher conformational freedom of the longer chains, which enhances binding of the particles to the surface of the mineral.^26^ Exceptions are noted, however, where longer sulfate chains were observed to give less incorporation than their shorter counterparts,^34^ even at low [Ca^2+^] = 1.5 mM.

The complexity of occluding anionic particles was further investigated by designing block copolymer micelles in which the ratio of carboxylate- to hydroxyl-functionalized chains could be systematically varied.^26^ Nanoparticle incorporation in calcite did not directly scale with the carboxylate content of the steric stabilizers, where nanoparticles comprising 1:1 carboxylate/hydroxyl groups were occluded at higher levels than those with carboxylate chains only. In this scenario, the hydroxyl moieties were considered to reduce ionic cross-linking between adjacent carboxylates chains, providing higher colloidal stability and more efficient binding to the crystal surface. These studies show that the incorporation of anionic particles in calcite is a complex process relying on trial and error to achieve success.

Cationic polyelectrolytes, in contrast, offer a straightforward and robust means of synthesizing hybrid crystals, where the weak interactions between the Ca^2+^ ions and cationic polymers ensure excellent colloidal stability of the nanoparticles. High concentrations of nanoparticles can therefore be employed. Although the cationic additives bind less strongly to the crystal surface than their anionic counterparts,^55^ access to much higher particle concentrations and supersaturations delivers exceptional occlusion levels: 57 vol % polymer nanoparticles, 64 wt % silver nanoclusters, and 70 wt % gold nanoparticles. This is achieved for a broad range of supersaturations and without compromising the single crystallinity of calcite, which enables scale-up. The polyamines employed were all commercially available, avoiding the need for the synthesis of bespoke polymers.

These results also contribute to the growing recognition that cationic additives can make valuable crystallization additives. While they have been largely neglected in the control of calcium carbonate and other inorganic crystals, some notable exceptions exist, such as the formation of fibers and thin films of CaCO_3_ in the presence of poly(allylamine hydrochloride) (PAH).^56,57^ Organic molecules bearing amine moieties,^58^ basic polypeptides,^59^ and cationic polyelectrolytes^40^ can additionally enable polymorph selection, delivering metastable vaterite and aragonite over calcite (the thermodynamically stable phase).^58,60,61^ Positively charged molecules may also play important roles in biogenic systems, where the analysis of the structures of mollusk shells has identified biomacromolecules rich in histidine, arginine, and lysine residues.^62−64^ Our demonstration that polyamines can drive the occlusion of particles to exceptionally high levels therefore further extends the scope of these molecules as crystallization additives.

## Conclusions

While cationic additives are often overlooked in favor of their anionic counterparts, growing evidence suggests that they can play pivotal roles in controlling the crystallization of inorganic compounds. This work demonstrates that functionalization of particulate additives with commercially available cationic polyelectrolytes offers a robust and versatile means of fabricating functional nanocomposites of a wide range of inorganic single crystals, including carbonates, sulfates, and oxides. Exceptional levels of occlusion of organic and inorganic particulate additives were achieved, far exceeding those obtained with their acidic counterparts. This work therefore provides a significant step-change in methodology, where we envisage that SERS combined with the intrinsic plasmonic properties of metal nanoparticles and nanoclusters within inorganic single crystals could find immediate applications in a wide range of areas, including catalysis, bioimaging, sensing, and photothermal therapy.

## Experimental Section

### Synthesis of the Positively Charged Particulate Additives

#### Synthesis of the PMMA-PEI Latex Nanoparticles

Poly(methyl methacrylate)-poly(ethyleneimine) (PMMA-PEI) core–shell nanoparticles were prepared by a one-step emulsion polymerization. Briefly, 1 g of branched PEI (*M*_W_ = 25 000 g mol^–1^) was dissolved in a round-bottomed flask containing 50 mL of DI water. The sealed reaction mixture was purged with N_2_ (g) for 1 h and was subsequently placed in an oil bath heated at 80 °C. Then, 2 g of MMA monomer were added to the solution under constant stirring (500 rpm) and a N_2_ (g) stream. After 15 min, TBHP (10 mM, 0.50 mL) was injected into the reaction solution. The reaction mixture was allowed to react at 80 °C for 3 h under an N_2_ (g) atmosphere. The product nanoparticles were purified by repeated centrifugations (5700 rcf, 30 min) to remove the unreacted reagents and were then redispersed in DI water.

#### Synthesis of the Au/PEI Nanoparticles

Au/PEI nanoparticles were synthesized by adding 2 mL of a 2 wt % HAuCl_4_·3H_2_O to 400 mL DI water containing 0.025 wt % PEI (*M*_W_ = 1200 g mol^–1^) under constant stirring (500 rpm). After 30 min, 5 mL of NaBH_4_ (20 mM) was injected into the solution. The solution was further stirred for 1 h and then subjected to multiple concentration/dilution cycles using an Amicon stirred ultrafiltration cell (Millipore) using a 10 kDa cellulose membrane and maintaining the pressure below 1 bar. The volume of the solution collected was adjusted to prepare a 1 wt % Au/PEI stock solution.

#### Synthesis of the Ag/PEI Nanoclusters

Ag/PEI nanoclusters were prepared by adding 250 μL of an aqueous AgNO_3_ solution (10 mM) dropwise to a 10 mL PEI (*M*_W_ = 10 000 g mol^**–**1^) solution (250 μM). The pH of the solution was subsequently adjusted to 4.5 using HCl (aq) (1 M). Then, 300 μL of l-ascorbic acid (100 mM) was injected into the solution. After 12 h, the solution was purified by dialysis against DI water (MWCO = 3500 Da, SpectraPor) and the nanoclusters were isolated by lyophilization. The freeze-dried Ag/PEI nanoparticles were then redispersed in DI water to give a 1 wt % Ag/PEI stock solution.

### CaCO_3_ Mineralization in the Presence of Positively Charged Additives

Calcium carbonate (CaCO_3_) was precipitated using the ammonium carbonate diffusion method^37^ in the presence of the basic additives (l-lysine, l-arginine, polymer nanoparticles, gold nanoparticles, and silver nanoclusters), the acidic polymer nanoparticles, or the nonfunctionalized polymer nanoparticles. Briefly, 1 mL of the prepared solution containing the desired amounts of additives and [Ca^2+^] = 1.5–20 mM were transferred to well plates containing glass substrates. Calcium carbonate was precipitated by placing the well plates in a desiccator along with a Petri dish containing 2 g of (NH_4_)_2_CO_3_. Crystallization was allowed to proceed overnight (>12 h). After this time, the substrates supporting the crystals were washed several times with DI water and then ethanol, followed by gentle drying using a N_2_ (g) stream.

### Mineralization of MnCO_3_, SrSO_4_, and CaSO_4_ in the Presence of Au/PEI Nanoparticles

MnCO_3_, SrSO_4_, and gypsum (CaSO_4_·2H_2_O) crystals were precipitated in the presence of Au/PEI nanoparticles by mixing 0.1–0.25 wt % of Au/PEI with an aqueous solution containing [Mn^2+^] = 2 mM, [Sr^2+^] = 2 mM or [Ca^2+^] = 100 mM, respectively. [NaHCO_3_] = 100 mM, [Na_2_SO_4_] = 10 mM, or [Na_2_SO_4_] = 100 mM were then added to the manganese, strontium, or calcium solutions, respectively. Crystallization reactions were allowed to proceed overnight (>12 h). The substrates supporting the crystals were then washed several times with DI water and then ethanol, followed by gentle drying using an N_2_ (g) stream prior to characterization.

### Synthesis of Au/PEI–ZnO Composite Crystals

A round-bottomed flask was charged with 2.5 mL of 1 wt % Au/PEI and an aqueous solution of zinc nitrate hexahydrate (1.50 mmol) to give a total volume of 97.5 mL. The reaction mixture was connected to a condenser and was placed in a preheated oil bath at 90 °C and stirred for 30 min. Crystallization of ZnO containing Au/PEI was initiated by the slow addition of a 2.5 mL aqueous solution of HMTA (1.50 mmol). The reaction was then allowed for 90 min and was quenched by immersing the flask in an ice bath. The composite crystals were then isolated by repeated centrifugations (2850 rcf, 10 min), washed with water and ethanol, and then dried in an oven (50 °C).

### Characterization

#### Dynamic Light Scattering (DLS) and Electrophoretic Analyses

The hydrodynamic diameters, particle size distributions (PDI), and ζ-potentials of the cationic nanoparticles were measured using a Malvern Zetasizer NanoZS at a fixed scattering angle of 173°. The colloidal stability of the PEI-functionalized nanoparticles and the carboxyl-functionalized nanoparticles was assessed by monitoring the evolution of the hydrodynamic diameters of the nanoparticles in solutions containing the nanoparticles (0.10 wt %) in [Ca^2+^] = 0–50 mM. The aqueous suspensions were adjusted to pH = 9 using 100 mM NaOH, which corresponds to the alkaline pH of the mineralization solution of CaCO_3_.

#### Electron Microscopy

The crystals were imaged with scanning electron microscopy (SEM) using an FEI NanoSEM Nova 450. The samples were mounted on SEM stubs using carbon adhesive disks and coated with a 4 nm iridium layer prior to imaging. Cross sections through the composite crystals were prepared using focused ion beam (FIB) milling with a FEI Helio G4 CX dual-beam high-resolution monochromated FEG SEM instrument equipped with a FIB. A selected area of the crystal was precoated with 2 μm thick Pt. The operating voltage was 30 kV, and the beam currents were varied between 0.1 and 5 nA.

Transmission electron microscopy (TEM) analyses of the cationic nanoparticles were carried out by placing a 10 μL droplet of an aqueous suspension of the nanoparticles (0.10 wt %) on a copper TEM grid-coated with a continuous carbon film for 1 min. TEM analyses were conducted using a FEI Tecnai TF20 FEGTEM with an Oxford Instruments INCA 350 EDX system/80 mm X-Max SDD detector and a Gatan Orius CCD camera operating at 200 kV.

Au/calcite and Ag/calcite composite crystals were characterized by TEM. Thin lamellae were prepared from the composite crystals using FIB-SEM and transferred to a copper TEM grid using a Kleindiek micromanipulator. The homogeneous incorporation of the cationic nanoparticles in calcite was confirmed using a high-angle annular dark-field scanning TEM (HAADF-STEM), in conjunction with EDX analysis mapping using a FEI Titan3 Themis G2S/TEM operated at 300 kV and 3 nA with a FEI Super-X energy dispersive X-ray (EDX) system and a Gatan OneView CCD camera.

#### Atomic Absorption Spectroscopy (AAS)

Quantification of the amount of Au nanoparticles incorporated within calcite single crystals was carried out using a PerkinElmer atomic absorption spectrometer AAnalyst 400 operating with an air-acetylene flame. The Au/calcite composite crystals were dissolved in 250 μL of concentrated *aqua regia* solution (HCl/HNO_3_ – 3:1 molar ratio), which was then diluted to 50 mL with DI water. The amount of elemental Au and Ca present in the sample was then measured after calibration using Au and Ca standard solutions.

#### Inductively Coupled Plasma-Optical Emission Spectroscopy (ICP-OES)

Quantification of the amount of Ag nanoclusters incorporated within calcite single crystals and Au nanoparticles incorporated within MnCO_3_, SrSO_4_, CaSO_4_, and ZnO was carried out using a Thermo Fisher Scientific iCAP 7400 radial ICP-OES Analyzer. The Ag/calcite composite crystals were dissolved in 250 μL of HNO_3_ solution (1 M) and then diluted to 50 mL with DI water. Au/MnCO_3_, Au/SrSO_4_, Au/CaSO_4_, and Au/ZnO were first dissolved in 250 μL of concentrated *aqua regia* solution, which was then diluted to 50 mL with DI water. The amount of elemental Ag, Au, Ca, Mn, Sr, and Zn present in the samples was then measured after calibration using Au, Ca, Mn, Sr, and Zn standard solutions.

#### Thermogravimetric Analysis (TGA)

Thermogravimetric analyses were performed from 20 to 850 °C in air, using a TA-Instruments Q600 operating at 10 °C min^**–**1^. The samples were bleached prior to characterization to remove the surface-bound organic matter.

#### Single-Crystal XRD

Au/calcite and Ag/calcite composite crystals were fixed to microloops using oil and mounted on a Rigaku XtaLAB Synergy Custom X-ray diffractometer (Cu Kα radiation *λ* = 1.54184 Å). Diffraction data were collected on a HyPix-6000HE hybrid photon counting (HPC) detector. The crystals were kept at 293 K during data collection, which was carried out for a 2*θ* range = 23.064–134.602°. Initial data collection, indexing, and integration procedures were performed using Rigaku Oxford Diffraction software, CrysAlisPro. The resulting data were solved and refined within Olex2^65^ with the ShelXT^66^ structure solution program using Intrinsic Phasing and refined with the ShelXL^67^ refinement package using Least-squares minimization.

#### Synchrotron High-Resolution PXRD

Pure calcite single crystals and calcite-incorporating PEI, Au/PEI nanoparticles, Ag/PEI nanoclusters, and PMMA-PEI latex particles were analyzed using HRPXRD on beamline ID22 at the European Synchrotron Research Facility (ESRF), Grenoble, France, at a wavelength of (0.354496 ± 0.000005) Å. Instrument calibration was carried out using a high-purity NIST SRM640c Si(111) standard. The instrumental contribution to the peak broadening does not exceed 0.003° (2*θ*), and peak positions are accurate and reproducible to a few tenths of a millidegree. The powder samples were loaded into 0.5 mm borosilicate glass capillaries, and the diffractograms were recorded at room temperature.

The structural parameters were refined by Rietveld analysis using PANalytical X’Pert HighScore Plus software. Lattice distortions, microstrain fluctuations, and coherence lengths (i.e., crystallite sizes) were measured for the whole spectra, and the (012), (104), (006), and (110) reflections of calcite using Rietveld and line profile analyses. The goodness of fit (GOF) for all analyzed samples was <8, showing the good quality of the fittings.

#### Other Measurements

Optical micrographs of the specimens were recorded using a Nikon Eclipse LV100 polarizing microscope equipped with both transmitted and reflected light sources. Fluorescence microscopy images of the Ag/PEI nanoclusters incorporated within calcite single crystals were recorded using a Zeiss Axio Scope A1 microscope fitted with an AxioCam monochrome camera light source. Individual crystal polymorphs were obtained by Raman spectroscopy using a Renishaw 2000 Raman Microscope equipped with a 785 nm diode laser. Spectra were recorded using 10 s exposure times and 5% laser power. Fourier transform infrared (FTIR) spectra were acquired over the mid-infrared region (600–2000 cm^–1^) using a PerkinElmer ATR-IR instrument. UV–visible extinction measurements of the Au/PEI nanoparticles dispersed in DI water and in the presence of [Ca^2+^] = 0–50 mM were carried out using a NanoDrop One/One^C^ Microvolume UV–Vis spectrophotometer.

## Supporting Information Available

Experimental details, including the synthesis and characterization of the polymer and metal nanoparticles, their incorporation in calcite and alternative host single crystals, additional optical micrographs, DLS, electrophoretic, UV–visible, Raman, HRPXRD, TGA, TEM-EDX, SEM-EDX, and FIB-SEM analyses, and supplementary information about the MD simulations (PDF). The data associated with this paper is available from University of Leeds at https://doi.org/10.5518/1146).
