# Supplementary material for: Positively Charged Additives Facilitate Incorporation in Inorganic Single Crystals
Source: Chem Mater. 2022 May 18;34(11):4910–23. doi: 10.1021/acs.chemmater.2c00097 (PMC9202304; doi:10.1021/acs.chemmater.2c00097)
Supplement: Supplementary file 1 — cm2c00097_si_001.pdf [file cm2c00097_si_001.pdf]

Positively Charged Additives Facilitate Incorporation in Inorganic Single Crystals

Ouassef Nahi,[1] Alexander Broad,[2] Alexander N. Kulak,[1] Helen M. Freeman,[3] Shuheng Zhang,[1] Thomas D. Turner,[1] Lucien Roach,[4] Robert Darkins,[2] Ian J. Ford,[2] and Fiona C. Meldrum[1]**

[1] School of Chemistry, University of Leeds, Woodhouse Lane, Leeds, LS2 9JT, UK

[2] Department of Physics and Astronomy, University College London, Gower Street, London WC1E 6BT, UK

[3] School of Civil Engineering, University of Leeds, Woodhouse Lane, Leeds, LS2 9JT, UK

[4] CNRS, Univ. Bordeaux, Bordeaux INP, ICMCB, UMR 5026, 33600 Pessac, FRANCE

S1. Materials

$CaCl_2{\cdot}2H_2O$, $MnCl_2{\cdot}4H_2O$, $SrCl_2{\cdot}6H_2O$, $Zn(NO_3)_2{\cdot}6H_2O$, $(NH_4)_2CO_3$, $NaHCO_3$, Na_2SO_4, hexamethylenetetramine (HMTA), gold (III) chloride trihydrate ($HAuCl_4{\cdot}3H_2O$, > 99%), sodium borohydride ($NaBH_4$), silver nitrate ($AgNO_3$, > 99%), L-lysine (> 98%), L-arginine (> 98%), L-ascorbic acid (> 98%), 8-Hydroxypyrene-1,3,6-trisulfonic acid trisodium salt (HPTS, > 96%), branched poly(ethyleneimine) (PEI) (M_W = 1,200 g mol^{-1}, 10,000 g mol^{-1} and 25,000 g mol^{-1}), methyl methacrylate (MMA) monomer (> 99%), and tert-butyl hydroperoxide (TBHP, 70 vol% solution in water) were purchased from Sigma Aldrich (UK) and were used as received. Carboxyl-functionalized latex particles (average diameter ≈ 200 nm) were purchased from Bangs Laboratories, Inc. (USA). Non-functionalized latex nanoparticles (average diameter ≈ 200 nm) were purchase from Thermo Fisher Scientific (UK). All solutions were prepared using Milli-Q deionized (DI) water. Glass slide substrates were thoroughly cleaned by soaking in piranha solution (H_2SO_4 : H_2O_2 – 70 vol% : 30 vol%), washed with DI water followed by ethanol, and dried using N_2(g) stream, prior to use.

S2. Synthesis of the positively charged particulate additives

S2.1. Synthesis of the PMMA-PEI latex nanoparticles

Poly(methyl methacrylate)-poly(ethyleneimine) (PMMA-PEI) core-shell nanoparticles were prepared by a one-step emulsion polymerization, according to a procedure reported elsewhere.[1] Briefly, 1 g of branched PEI (M_W = 25,000 g mol^{-1}) was dissolved in a round-bottomed flask containing 50 mL of DI water. The sealed reaction mixture was purged with N_2(g) for 1 h and was subsequently placed in an oil bath heated at 80°C. After temperature equilibration, 2 g of MMA monomer was slowly added to the solution under constant stirring and N_2(g) stream. After 15 min, TBHP (10 mM, 0.50 mL) was injected in the reaction solution. The sealed reaction mixture was allowed to react at 80°C for 3 h under N_2(g) atmosphere. The product nanoparticles were then purified by repeated centrifugations (5,700 rcf, 30 min) to remove the unreacted reagents. The latex particles were then redispersed in DI water.

S2.2. Synthesis of the Au/PEI nanoparticles

5 nm Au/PEI nanoparticles were synthesized by adding 2 mL of a 2 wt% $HAuCl_4 \cdot 3H_2O$ to 400 mL DI water containing 0.025 wt% PEI (M_W = 1,200 g mol^{-1}) under constant stirring (500 rpm). The solution immediately turned bright orange, indicating complexation between the gold ions and PEI. After 30 min, 5 mL of $NaBH_4$ (20 mM) was injected into the solution. The reaction mixture gradually turned ruby red, indicative of the formation of Au nanoparticles. The solution was further stirred for 1 h and then subjected to multiple concentration/dilution cycles using an Amicon® stirred ultrafiltration cell (Millipore) using a 10 kDa cellulose membrane and maintaining the pressure below 1 bar. The volume of solution collected was adjusted to prepare a 1 wt% Au/PEI stock solution.

S2.3. Synthesis of the Ag/PEI nanoclusters

Ag/PEI nanoclusters were prepared using an existing protocol.[2] Briefly, 250 µL of an aqueous $AgNO_3$ solution (10 mM) was added dropwise to a 10 mL PEI (M_W = 10,000 g mol^{-1}) solution (250 µM) under constant stirring (500 rpm). The pH of the solution was subsequently adjusted to 4.5 using HCl(aq) (1 M). 300 µL of L-ascorbic acid (100 mM) was then injected into the solution. After completion of the reaction (12 h), the solution was purified by dialysis against DI water (MWCO = 3500 Da, SpectraPor) and the particles were isolated by lyophilization. The freeze-dried Ag/PEI nanoparticles were then redispersed in DI water to give a 1 wt% Ag/PEI stock solution.

S3. $CaCO_3$ mineralization in the presence of positively charged additives

Calcium carbonate ($CaCO_3$) was precipitated using the ammonium carbonate diffusion method[3] in the presence of the basic additives (L-lysine, L-arginine, polymer nanoparticles, gold nanoparticles and silver nanoclusters), the acidic polymer nanoparticles or the non-functionalized polymer nanoparticles. The glass substrates were placed at the bottom of multi-well plates. 1 mL of prepared solutions containing desired amounts of additives and $[Ca^{2+}]$ = 1.5 mM – 20 mM were then transferred to the well plates. Calcium carbonate was precipitated by placing the well plates in a desiccator along with a petri-dish containing 2 g of $(NH_4)_2CO_3$ that was covered with Parafilm and punctured several times with a needle. Crystallization was allowed to proceed overnight (> 12 h). After this time the substrates supporting the crystals were washed several times with DI water and then ethanol, followed by gentle drying using N_2(g) stream.

S4. Mineralization of $MnCO_3$, $SrSO_4$ and $CaSO_4$ in the presence of Au/PEI nanoparticles

$MnCO_3$, $SrSO_4$ and gypsum ($CaSO_4.2H_2O$) crystals were precipitated in the presence of Au/PEI nanoparticles by mixing 0.1 wt% of Au/PEI with an aqueous solution containing $[Mn^{2+}]$ = 2 mM, $[Sr^{2+}]$ = 2 mM or $[Ca^{2+}]$ = 100 mM respectively. $[NaHCO_3]$ = 100 mM, $[Na_2SO_4]$ = 10 mM or $[Na_2SO_4]$ = 100 mM were then added to the manganese, strontium or calcium solutions, respectively. Homogeneous incorporation of the gold nanoparticles throughout the gypsum crystals was achieved by increasing the Au/PEI concentration to 0.25 wt%. Crystallization reactions were allowed to proceed overnight (> 12 h). The substrates supporting the crystals were then washed several times with DI water and then ethanol, followed by gentle drying using N_2(g) stream, prior to characterization.

S5. Synthesis of Au/PEI – ZnO composite crystals

A round-bottomed flask (RBF) was charged with 2.5 mL of 1 wt% Au/PEI and an aqueous solution of zinc nitrate hexahydrate (1.50 mmol) to give a total volume of 97.5 mL. The reaction mixture was connected to a condenser and was placed in a pre-heated oil bath at 90°C and stirred for 30 min. Crystallization of ZnO containing Au/PEI was initiated by slow addition of a 2.5 mL aqueous solution of HMTA (1.50 mmol) under vigorous stirring (500 rpm). The reaction was then allowed for 90 min and was quenched by immersing the RBF in an ice bath. The composite crystals were then isolated by repeated centrifugations (2850 rcf, 10 min), washed with water and ethanol, and then dried in an oven (50°C).

S6. Finite element modelling of SERS

The optical responses of the dimers, trimers and tetramers were simulated using COMSOL's radio frequency module. Calculations were performed in the frequency domain in the scattered field formulation using the PARDISO direct solver. Refractive index values for Au were taken from Johnson & Christy.[4] Refractive index values for calcite were taken from Ghosh.[5] Particles were simulated by embedding them within calcite surrounded by a perfectly matched layer. σ_{abs} was calculated through a volume integral of the resistive heat losses inside all particles, Q_{rh}. σ_{scat} was calculated through a surface integral of the Poynting vector, over the surfaces of all particles.

S7. Molecular dynamics simulations of the binding of cationic additives to calcite

Molecular dynamics simulations were performed using LAMMPS[6] with a timestep of 1 fs. The temperature of the simulation was kept at 300 K using a Nosé-Hoover thermostat with a relaxation time of 100 fs. During equilibration, zero pressure was achieved using a Nosé-Hoover barostat with a relaxation time of 1000 fs. All long-range electrostatics were handled using a PPPM method with an accuracy of 10^{-4}. Periodic boundaries were used in all directions. The interactions for calcium carbonate, including their interactions with water, were described by the force fields of Raiteri *et al.*,[7] which are fitted to solvation free energies. The self-interactions of water were described by SPC/Fw.[8] Finally, the force fields for all additives and their interactions with calcite and water were taken from the General AMBER Force Fields (GAFF).[9] The partial charges were obtained using ANTECHAMBER.[10]

A slab of calcite that was periodic in the x and y-directions was placed within the simulation box. A 4 nm gap (excluding the partial layers) divided each slab with its periodic image in the z-direction. This gap was filled with water and the relevant additives. For simulations involving terraces or steps, the box sizes corresponded to 12 repetitions of the calcium carbonate unit cell in the x- and y-directions. For simulations involving kink sites, the box sizes corresponded to 12 repetitions in the x-direction and 13 in the y-direction. In this instance, the monoclinic skew of the simulation box was adjusted to allow for the periodicity of the crystal and the existence of a kink site. In each instance, the z-length was given by a 4 nm gap plus the 5 repetitions of the crystal unit cell. For each configuration, the z-dimension of the simulation box was relaxed under zero pressure for 1 ns to obtain the average length which was then fixed. All subsequent simulations were performed in the NVT ensemble after a 100 ps equilibration. In each

simulation, the total momentum of the calcite crystal was eliminated at every timestep to prevent drift during the simulation.

For simulations of step sites, the acute step structure was chosen since amine groups are found to preferentially bind to acute sites. For this step, only two distinct carbonate sites exist for the amines to bind to, and these can be included in a single simulation. However, for simulations involving kink sites, a total of four carbonate-terminated sites exist which cannot be represented in a single small simulation cell. However, calculations of dissolution enthalpies[11] show that one such site has a significantly higher dissolution enthalpy than the other. This does not guarantee that the dissolution free energy is highest, but we can make an educated guess that this kink site will be the most strongly bound and will therefore be the dominant exposed carbonate-terminated kink site. For this reason, this site was chosen for all simulations involving kink sites. The carbon atom in the terminating carbonate ion was restrained to its initial position in all dimensions using a harmonic potential with a spring constant of 50 kJ mol^{-1} $Å^{-2}$. This was done to ensure the stability of the kink site throughout the simulations. Rather than model the complete amino acids, we opted for the more tractable approach of isolating the relevant side chains, requiring fewer reaction coordinates.

S7.1. Binding free energies

Binding free energies were calculated for terrace, step and kink sites using metadynamics as implemented in PLUMED[12]. In all simulations, the reaction coordinate was taken as the position of the nitrogen atom in the amine group. For the terrace, only the z-position of the nitrogen atom was biased. For the steps and kinks, a preliminary metadynamics simulation explored all three coordinates (x, y and z) of the amine to identify the position of the thermodynamic minimum.

A subsequent metadynamics simulation was performed, where the in-plane (x,y)-position of the nitrogen atom was constrained to the (x,y)-position of the thermodynamic minimum and only the z-direction was used as a collective variable. For the three-dimensional simulations, Gaussians of height $k_BT/2$ and widths of 0.5 Å in all dimensions were deposited every ps. For the one-dimensional simulations, Gaussians of height $k_BT/2$ and widths 0.1 Å were deposited every 0.5 ps. In all cases, the multiple walkers algorithm[13] was applied to 16 parallel simulations communicating every 10 ps. Well-tempering[14] was also employed in the latter (one-dimensional) simulations. The position of the nitrogen atom was confined to the sampled

region *via* a set of upper and lower walls applying a harmonic force with spring constant 100 kJ(mol)$^{-1}$Å^{-2}.

S8. Characterization methods

S8.1. Dynamic light scattering (DLS) and electrophoretic analyses

The hydrodynamic diameters, particle size distributions (PDI) and zeta potentials of the Au/PEI, Ag/PEI, and latex nanoparticles were measured using dynamic light scattering (DLS) and electrophoretic analyses. The measurements were carried out using a Malvern Zetasizer NanoZS at a fixed scattering angle of 173°. The colloidal stability of the PEI-functionalized nanoparticles and the carboxyl-functionalized nanoparticles was assessed by monitoring the evolution of the hydrodynamic diameters of the nanoparticles in solutions containing the nanoparticles (0.10 wt%) in [Ca^{2+}] = 0 – 50 mM. The aqueous suspensions were adjusted to pH = 9 using 100 mM NaOH, which corresponds to the alkaline pH of the mineralization solution of $CaCO_3$.

S8.2. Electron microscopy

The crystals were imaged with scanning electron microscopy (SEM) using a FEI NanoSEM Nova 450. The samples were mounted on SEM stubs using carbon adhesive discs and coated with a 4 nm iridium layer, prior to imaging. Cross-sections through the composite crystals were prepared using focused ion beam (FIB) milling with a FEI Helio G4 CX dual beam-high resolution monochromated FEG SEM instrument equipped with a FIB. A selected area of the crystal was pre-coated with 2 μm thick Pt. The operating voltage was 30 kV and the beam currents were varied between 0.1 nA and 5 nA. SEM-EDX (energy dispersive X-ray) of the Ag/calcite composites revealed the efficient incorporation of the Ag nanoclusters in the calcite matrix.

Transmission electron microscopy (TEM) analyses of the Ag/PEI nanoclusters, Au/PEI nanoparticles and PMMA-PEI latex nanoparticles were carried out by placing a 10 μL droplet of an aqueous suspension of the nanoparticles (0.10 wt%) on a TEM grid for 1 min. Excess solution was removed *via* blotting. Copper TEM grids coated with a continuous carbon film were employed, and these were treated with a plasma glow discharge for 30 s to create a hydrophilic surface prior to addition of the aqueous solutions containing the nanoparticles. TEM analyses were conducted using a FEI Tecnai TF20 FEGTEM with an Oxford Instruments

INCA 350 EDX system/80 mm X-Max SDD detector and a Gatan Orius CCD camera operating at 200 kV.

Au/calcite and Ag/calcite composite crystals were characterized by TEM. Thin lamellae were prepared from the composite crystals using FIB-SEM and transferred to a copper TEM grid using a Kleindiek micromanipulator. The homogeneous incorporation of the Au/PEI nanoparticles in calcite was confirmed using a high-angle annular dark-field scanning TEM (HAADF-STEM), in conjunction with EDX analysis mapping of Ca/C (*i.e.,* $CaCO_3$) and Au/N (*i.e.,* Au/PEI), and imaging of the Ag/calcite composites were carried out using a FEI Titan3 Themis G2 S/TEM operated at 300 kV and 3 nA with a FEI Super-X energy dispersive X-ray (EDX) system and a Gatan OneView CCD camera.

S8.3. Atomic absorption spectroscopy (AAS)

Quantification of the amount of Au nanoparticles incorporated within calcite single crystals was carried out using AAS with a Perkin Elmer atomic absorption spectrometer AAnalyst 400, operating with an air-acetylene flame. The Au/calcite composite crystals were dissolved in 250 µL concentrated *aqua regia* solution (HCl : HNO_3 – 3:1 molar ratio), which was then diluted to 50 mL with DI water. The amount of elemental Au and Ca present in the sample was then measured after calibration using Au and Ca standard solutions.

S8.4. Inductively coupled plasma-optical emission spectroscopy (ICP-OES)

Quantification of the amount of Ag nanoclusters incorporated within calcite single crystals and Au nanoparticles incorporated within $MnCO_3$, $SrSO_4$, $CaSO_4$ and ZnO was carried out using a Thermo Fisher Scientific iCAP 7400 radial ICP-OES Analyzer. The Ag/calcite composite crystals were dissolved in 250 µL of HNO_3 solution (1 M), then diluted to 50 mL with DI water. Au/$MnCO_3$, Au/$SrSO_4$, Au/$CaSO_4$ and Au/ZnO were first dissolved in 250 µL concentrated *aqua regia* solution, which was then diluted to 50 mL with DI water. The amount of elemental Ag, Au, Ca, Mn, Sr and Zn present in the sample were then measured after calibration using Au, Ca, Mn, Sr and Zn standard solutions.

S8.5. Thermogravimetric analysis (TGA)

Thermogravimetric analyses were performed from 20°C to 850°C in air, using a TA-Instruments Q600 operating at 10°C min^{-1}. The samples were bleached prior to characterization to remove the surface bound organic matter.[15] Calcination of the pure calcite

crystals shows an onset of decomposition at 650°C, giving a weight loss of 44.0 wt% that is ascribed to the release of CO_2(g), leaving a residue of 56.0 wt% corresponding to CaO(s). Pyrolysis of the $PEI_{1,200}$/calcite hybrid crystals showed a weight loss of 16 wt% below 650°C due to the thermal decomposition of PEI incorporated within calcite. If the calcite crystals fully decompose at 800°C into CO_2(g) + CO(s), then an excess of ≈ 1.9 wt% of polymer remains in the crucible alongside the CO(s) residues. This most likely corresponds to the remaining PEI that does not fully decompose on annealing. Overall, this equates to ≈ 18 wt% of PEI incorporated within calcite single crystals.

Thermal decomposition of the PMMA-PEI latex particles/calcite crystals showed a weight loss of 20 wt% between room temperature and 650°C. If all calcite decomposes at 800°C, an excess of 12.9 wt% organic matter remains in the crucible, which is most likely attributed to the latex particles that do not fully decompose. This is confirmed by TGA analysis of the latex particles alone, where 15 wt% remained in the crucible, even after annealing at 850°C. This equates to 32.9 wt% of latex particles incorporated within calcite and corresponds to ≈ 57 vol% of the composite materials, based on the latex nanoparticle density of 1 g cm^{-3}. In comparison, no weight loss below 650°C was recorded for calcite precipitated in the presence of non-functionalized latex nanoparticles, which shows that they are not incorporated within the calcite crystals.

Annealing measurements of the Au/PEI nanoparticles and Ag/PEI nanoclusters show the presence of 65 wt% and 70 wt% of PEI polymer on the surface of the gold and silver particles, respectively. Considering that 42.5 wt% (AAS analysis) of Au is occluded within calcite and that these nanoparticles are surface-functionalized with 65 wt% of PEI (TGA analysis), this equates to ≈ 70.1 wt% of Au/PEI incorporated in calcite. Based on the density of PEI (1 g cm^{-3}), Au (19.3 g cm^{-3}) and calcite (2.7 g cm^{-3}), 6.5 vol% of Au and 63.5 vol% PEI are incorporated within calcite. Similarly, considering that 37.5 wt% of Ag clusters (9.5 vol%) are incorporated within calcite (ICP-OES analysis) and that these nanoclusters are surface-functionalized with 70 wt% of PEI (55.5 vol%), this represents ≈ 63.8 wt% (65 vol%) of Ag/PEI incorporated within calcite.

S8.6. Single-crystals XRD

Au/calcite and Ag/calcite composite crystals were fixed to micro-loops using an oil and mounted on a Rigaku XtaLAB Synergy Custom X-ray diffractometer (Cu-Kα radiation λ =

1.54184 Å) and diffraction data were collected on a HyPix-6000HE hybrid photon counting (HPC) detector. The crystals were kept at 293 K during data collection, which were carried out for a 2θ range = 23.064° - 134.602°. Initial data collection, indexing and integration procedures were performed within the Rigaku Oxford Diffraction software; CrysAlisPro. The resulting data were solved and refined within Olex2,[16] with the ShelXT[17] structure solution program using Intrinsic Phasing and refined with the ShelXL[18] refinement package using Least Squares minimization.

S8.7. Synchrotron High-Resolution PXRD

Pure calcite single crystals and calcite incorporating high levels of PEI, Au/PEI nanoparticles, Ag/PEI nanoclusters and PMMA-PEI latex particles were analyzed using synchrotron High-Resolution Powder X-Ray Diffraction (HR-PXRD) on beamline ID22 at the European Synchrotron Research Facility (ESRF), Grenoble, France, at a wavelength of (0.354496 ± 0.000005) Å. Instrument calibration was carried out using a high purity NIST SRM640c Si(111) standard. The instrumental contribution to the peak broadening does not exceed 0.003° (2θ), and peak positions are accurate and reproducible to a few tenths of a millidegree. The powder samples were loaded into 0.5 mm borosilicate glass capillaries and the diffractograms were recorded at room temperature.

The structural parameters were refined by Rietveld analysis using PANalytical X'Pert HighScore Plus software. Lattice distortions, microstrain fluctuations and coherence lengths (*i.e.,* crystallite sizes) were measured for the whole spectra, and the (012), (104), (006) and (110) reflections of calcite using Rietveld and line profile analyses. The goodness of fit (GOF) for all analyzed samples was < 8, showing the good quality of the fittings.

S8.8. Other measurements

Optical micrographs of the specimens were recorded using a Nikon Eclipse LV100 polarizing microscope, equipped with both transmitted and reflected light sources. Fluorescence microscopy images of the Ag/PEI nanoclusters incorporated within calcite single crystals were recorded using a Zeiss Axio Scope A1 microscope fitted with an AxioCam monochrome camera light source. Individual crystal polymorphs were obtained by Raman spectroscopy, using a Renishaw 2000 Raman Microscope, equipped with a 785 nm diode laser. Spectra were recorded using 10 s exposure times and 5% laser power. Fourier transform infrared (FTIR) spectra were acquired over the mid infrared region (600 cm^{-1} – 2000 cm^{-1}) using a Perkin–

Elmer ATR-IR instrument. UV-Visible extinction measurements of the Au/PEI nanoparticles dispersed in DI water and in the presence of [Ca^{2+}] = 0 – 50 mM were carried out using a NanoDrop One/One^{C} Microvolume UV-Vis spectrophotometer.

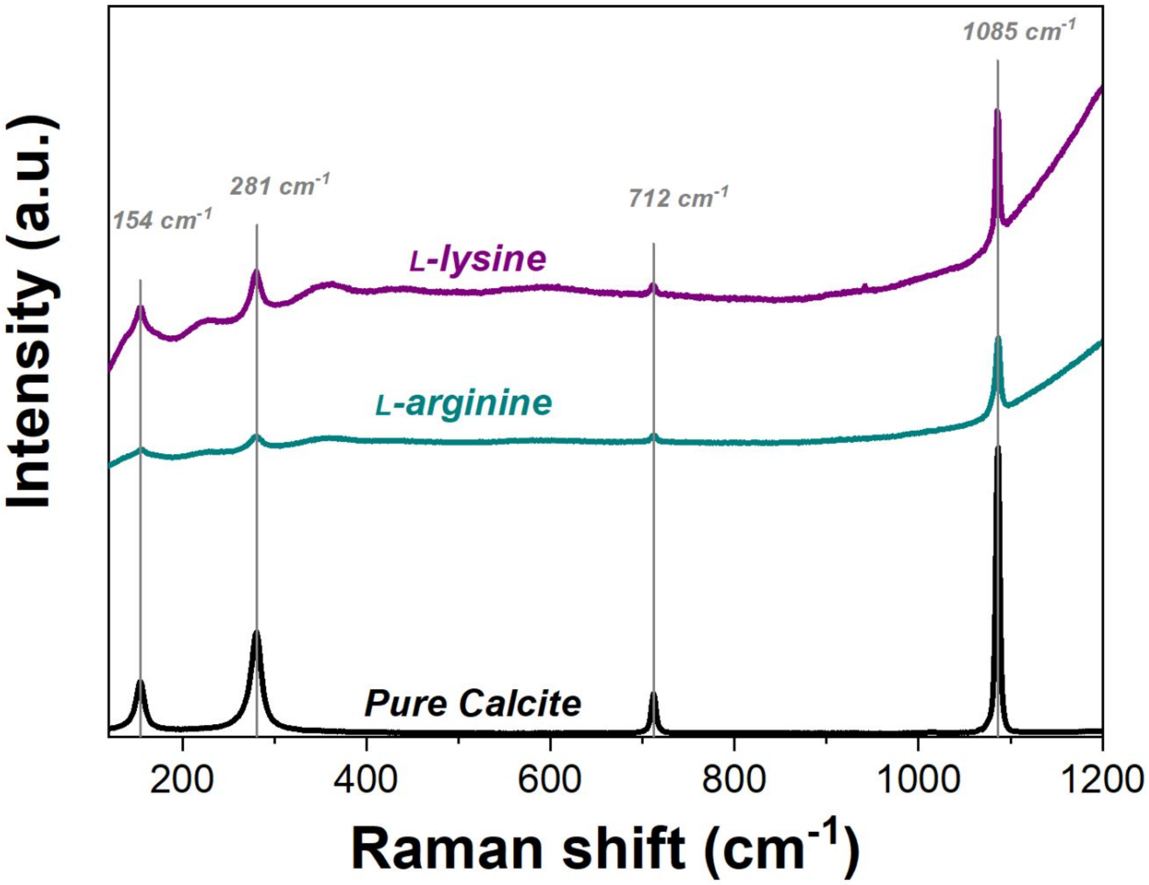

Figure S1. Raman spectra of $CaCO_3$ crystals precipitated in the presence of L-arginine (cyan), and L-lysine (purple). All crystals display the characteristic peaks: ν_1 (1085 cm^{-1}), ν_4 (712 cm^{-1}) and lattice modes (154 cm^{-1} and 281 cm^{-1}) of calcite (black).

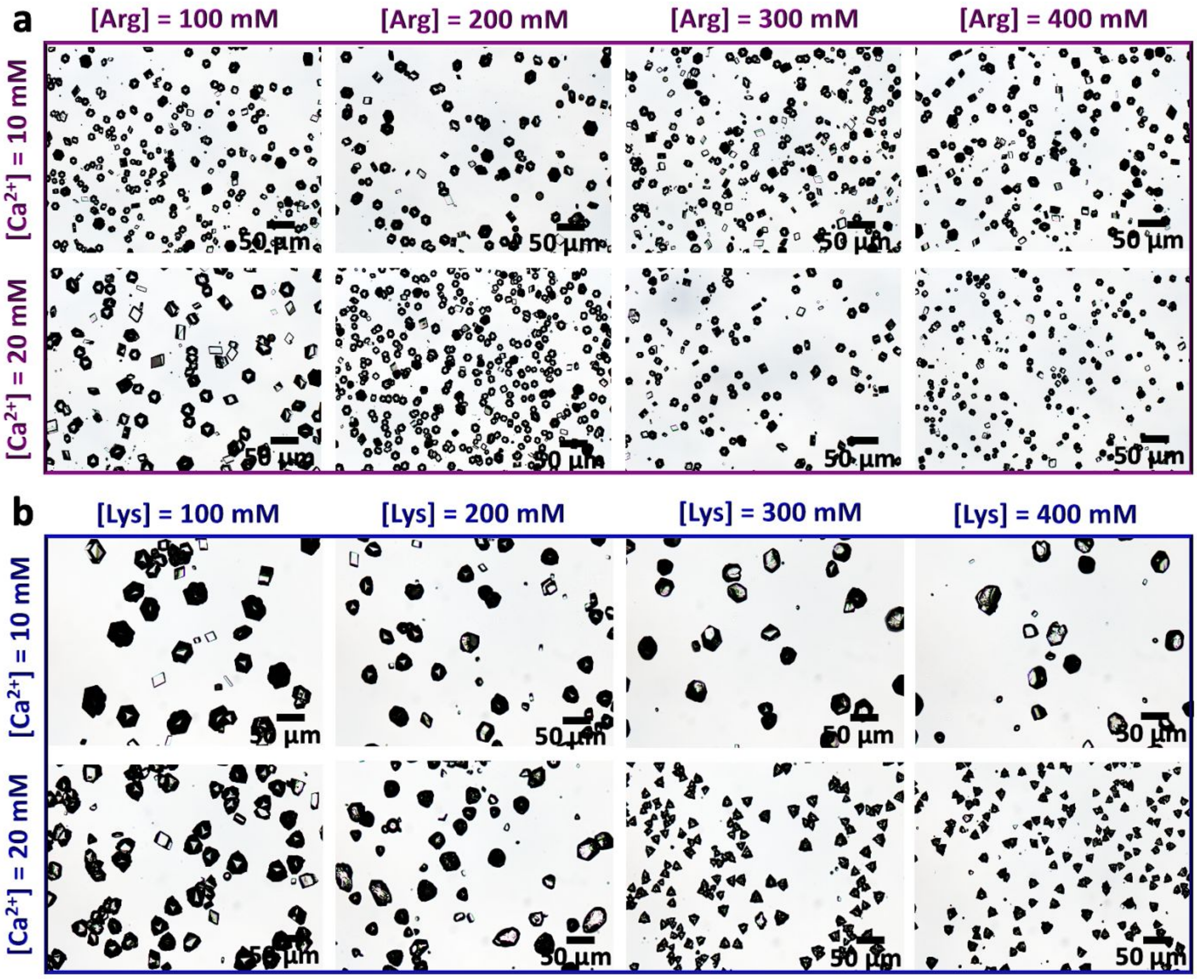

Figure S2. Calcite crystals precipitated in the presence of 100 – 400 mM of (a) L-arginine (Arg) and (b) L-lysine (Lys) in a mineralization solution containing [Ca^{2+}] = 10 mM – 20 mM. Only minor morphological changes are observed for Arg, whereas increasing the amount of Lys and/or [Ca^{2+}] in solution induces gradual elongation of the crystals along their *c*-axes.

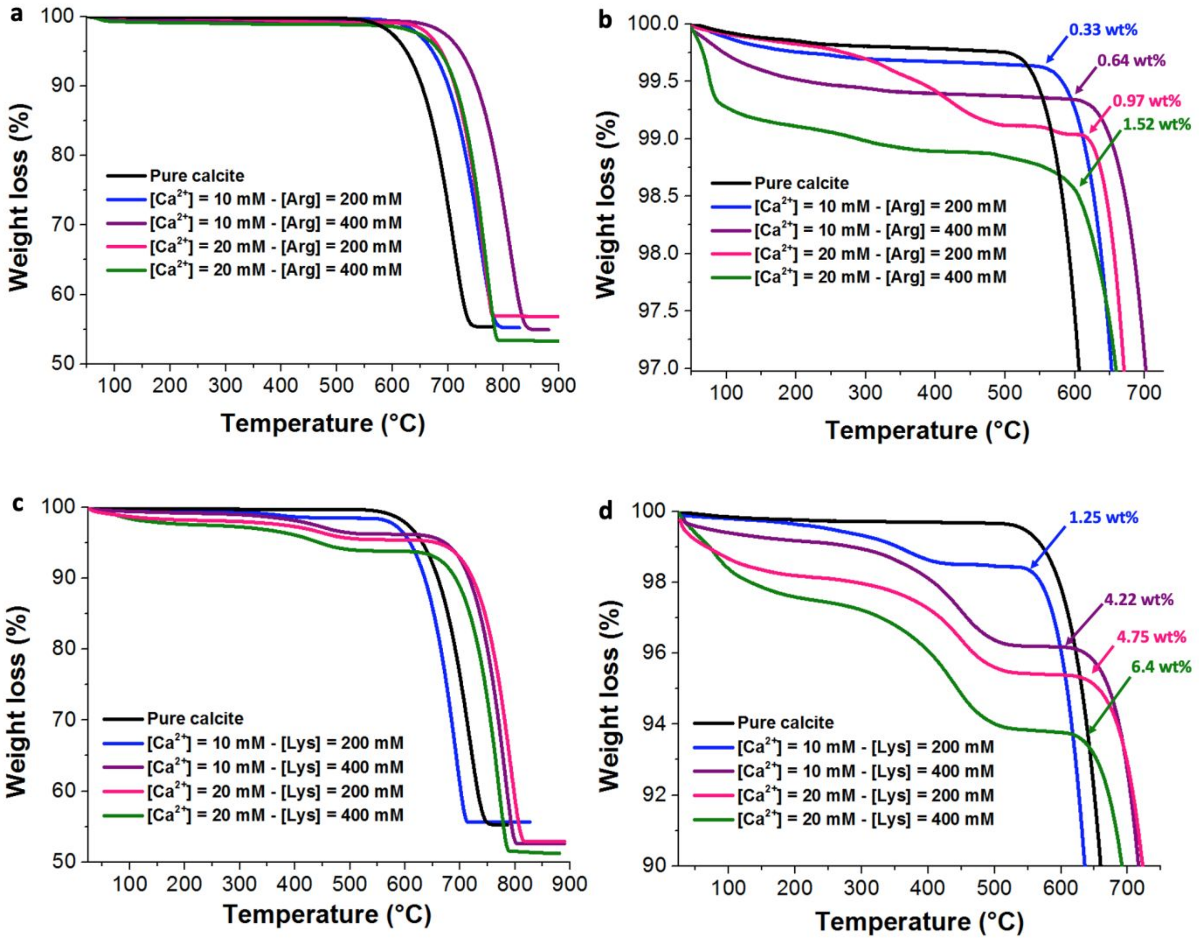

Figure S3. Thermogravimetric analyses (TGA) recorded in air for pure calcite (black), and calcite crystals incorporating (a-b) L-arginine (Arg) and (c-d) L-lysine (Lys) that were grown under the conditions indicated. The weight losses between room temperature and 700 °C correspond to the levels of amino acids incorporated (by weight) within calcite that are thermally decomposed.

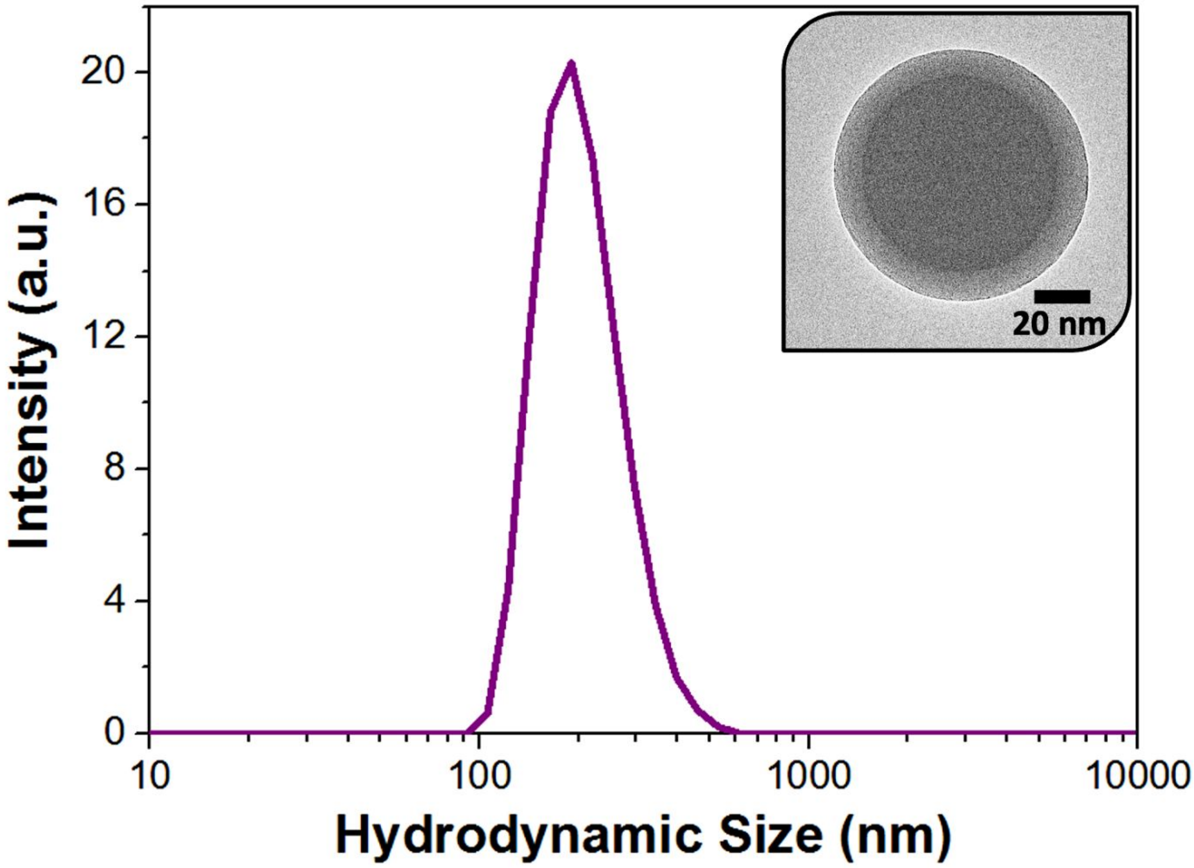

Figure S4. Size distribution analysis of the poly(methyl methacrylate)-poly(ethyleneimine) (PMMA-PEI) latex nanoparticles aqueous suspension (0.10 wt%) obtained by dynamic light scattering (DLS). The nanoparticles have unimodal diameter of ≈ 200 nm, and narrow size distributions, as indicated by their low polydispersity indices (PDI = 0.05).

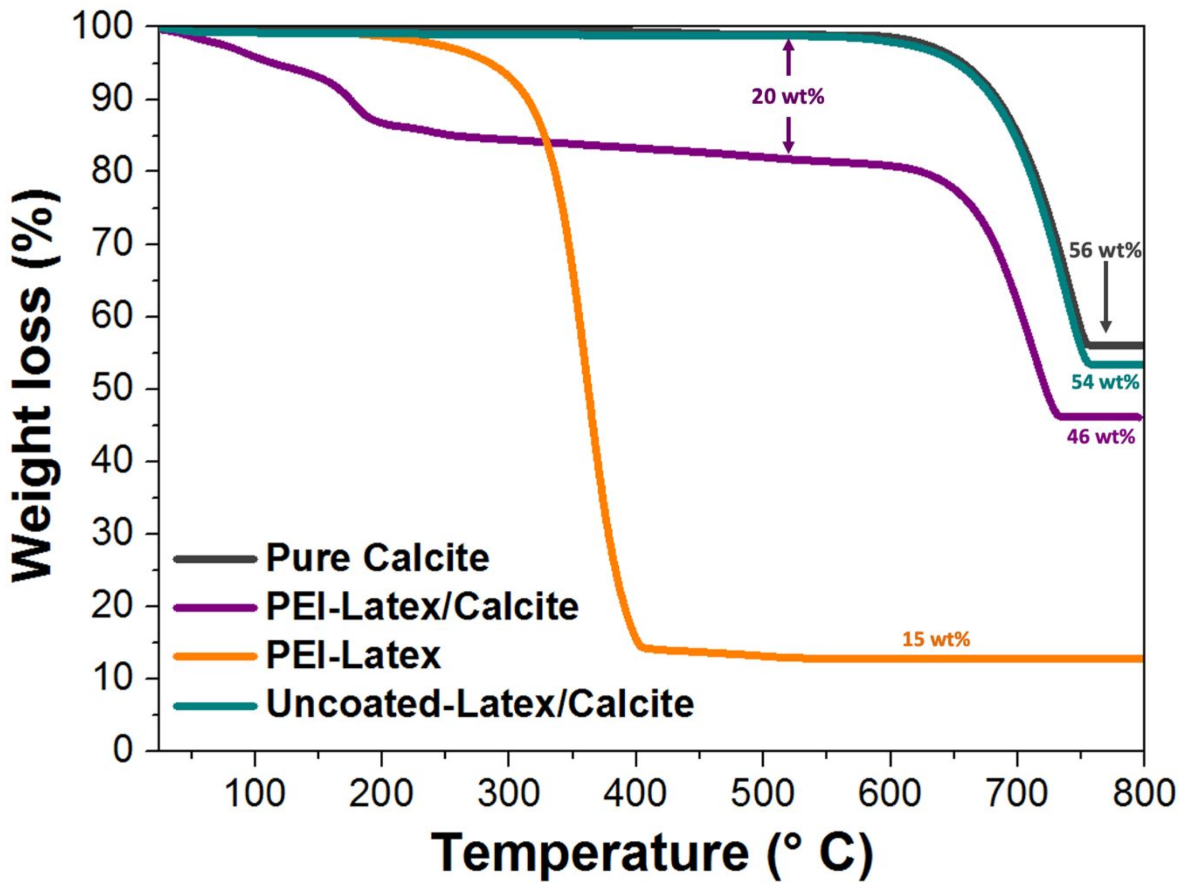

Figure S5. Thermogravimetric analyses (TGA) recorded in air for pure calcite (dark grey), and calcite crystals grown in the presence of [Ca^{2+}] = 10 mM and 0.5 wt% of PMMA-PEI latex nanoparticles (PEI-Latex/calcite, purple), non-functionalized latex nanoparticles (uncoated latex/calcite, cyan), and PMMA/PEI latex nanoparticle alone (orange). The weight losses between room temperature and 650°C correspond to the levels of nanoparticles incorporated within calcite that are thermally decomposed. A high level of incorporation of cationic latex particles (PMMA/PEI latex) is achieved, whereas no incorporation is recorded for the non-functionalized particles.

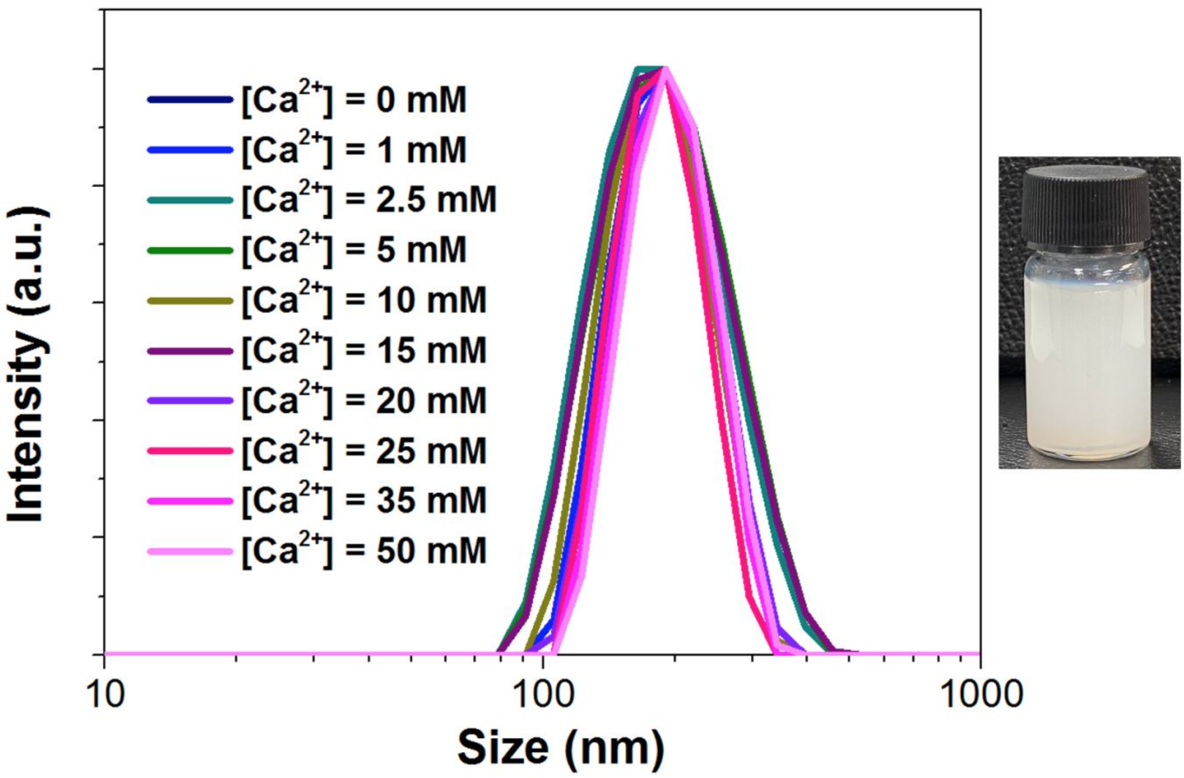

Figure S6. Size distribution analyses of the PMMA/PEI latex nanoparticles (0.10 wt%) using DLS. The nanoparticles are highly stable in solutions containing a broad range of $[Ca^{2+}]$ = 0 – 50 mM (pH = 9), as shown by the unimodal and narrow size distributions of the peaks. Image on the right is a solution containing the latex nanoparticles and $[Ca^{2+}]$ = 50 mM. No aggregation of the particles is observed, demonstrating excellent colloidal stability.

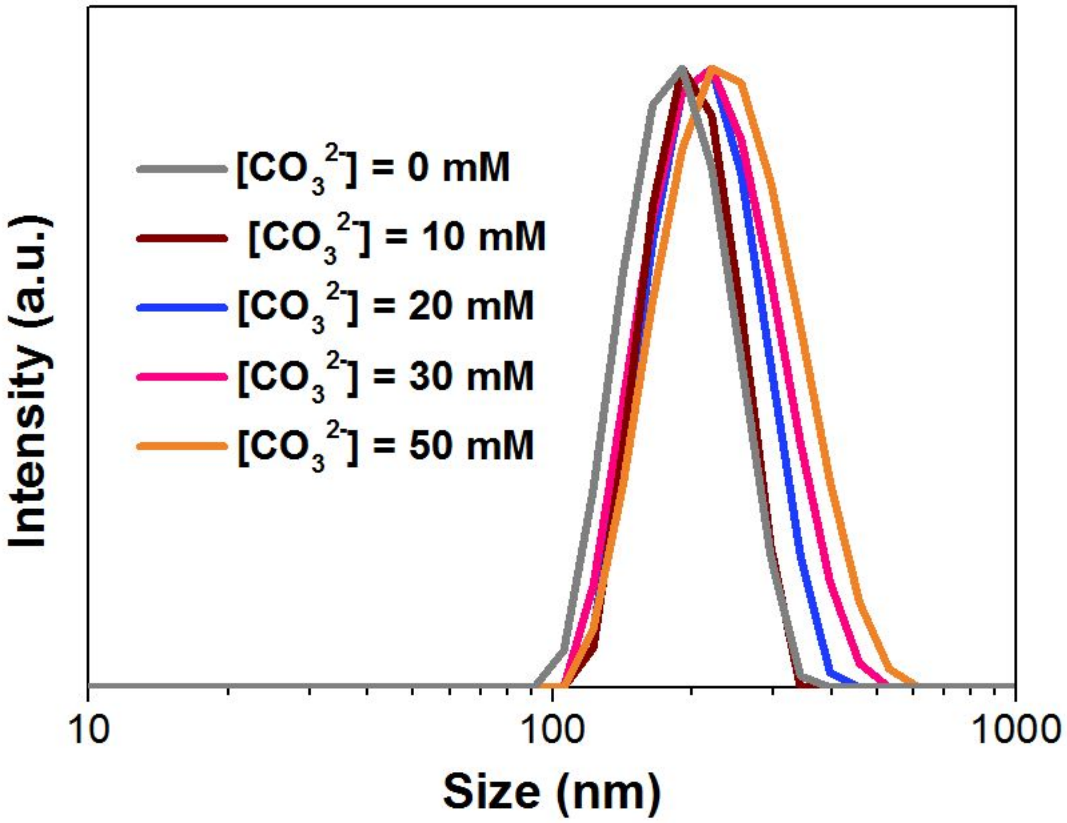

Figure S7. Size distribution analyses of the PMMA/PEI latex nanoparticles (0.10 wt%) using DLS. The nanoparticles are highly stable in solutions containing a broad range of $[CO_3^{2-}]$ = 0 – 50 mM (using Na_2CO_3), as shown by the unimodal and narrow size distributions of the peaks.

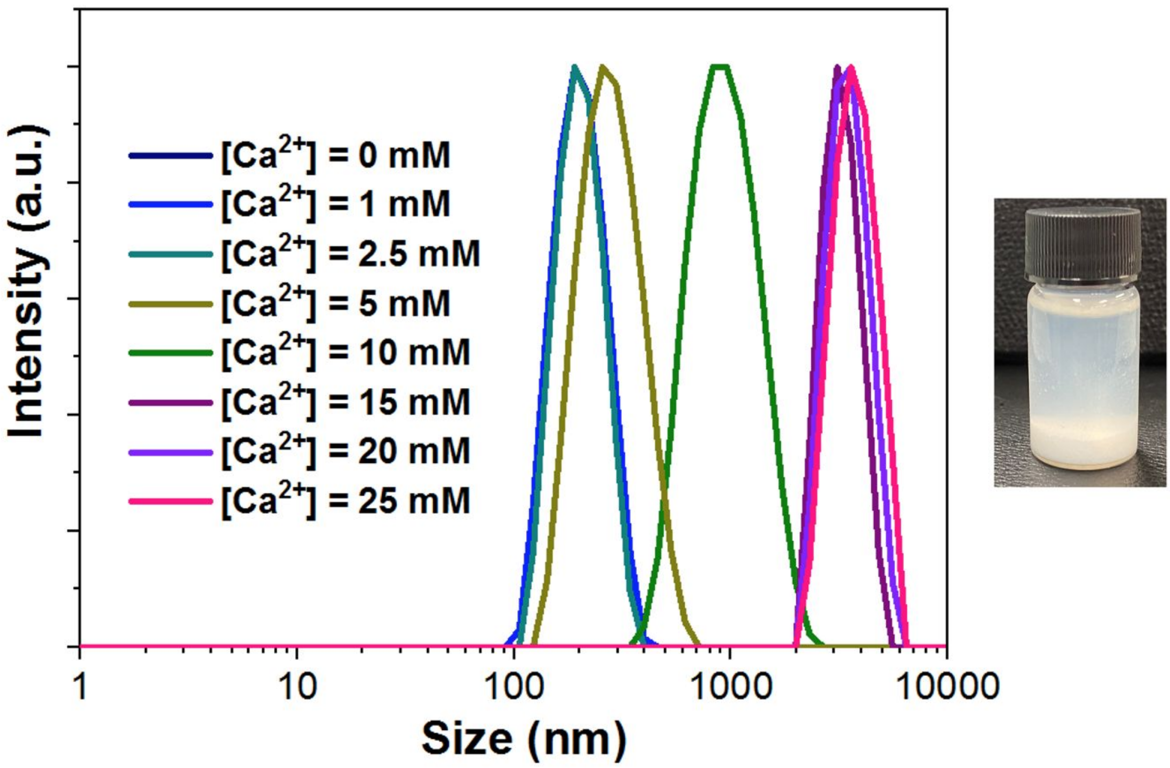

Figure S8. Size distribution analysis of the carboxyl-functionalized latex nanoparticles (0.10 wt%) using DLS. A gradual shift of the peaks toward higher hydrodynamic sizes can be observed as the amount of [Ca^{2+}] is increased from 0 – 25 mM (pH = 9), which indicates colloidal instability in solution. Aggregation of the nanoparticles is observed for [Ca^{2+}] > 5 mM. Image on the right is a solution containing the latex nanoparticles and [Ca^{2+}] = 10 mM. Severe aggregation and settling of the nanoparticles is observed.

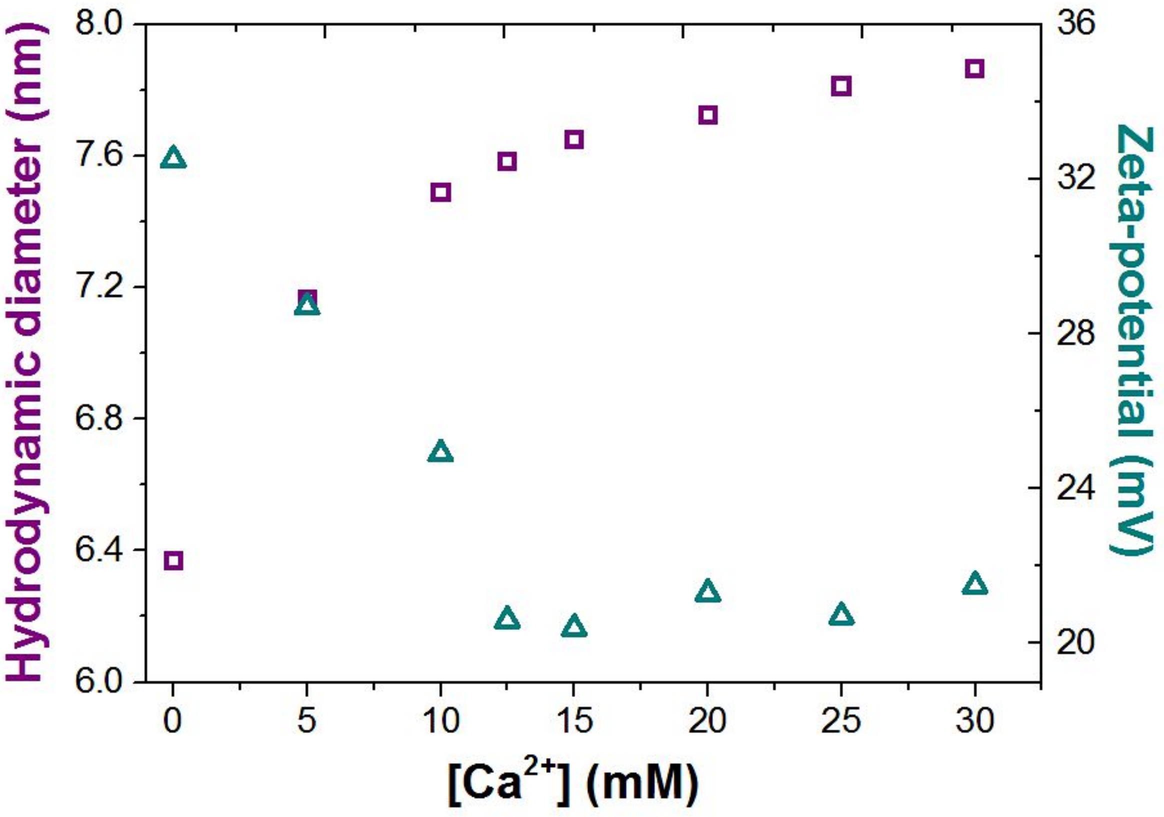

Figure S9. Evolution of the hydrodynamic diameter and zeta-potential of the Au/PEI nanoparticles in aqueous solutions containing $[Ca^{2+}]$ = 0 – 30 mM. The suspensions were adjusted to pH = 9 using 100 mM NaOH. Increasing $[Ca^{2+}]$ in solution only generates a minor increase of the Au/PEI hydrodynamic diameter, potentially due to the expansion of the PEI polymer on the surface of the Au particles. The nanoparticles remain highly positively charged in all conditions explored, which enables excellent colloidal stability in solutions containing a wide range of $[Ca^{2+}]$.

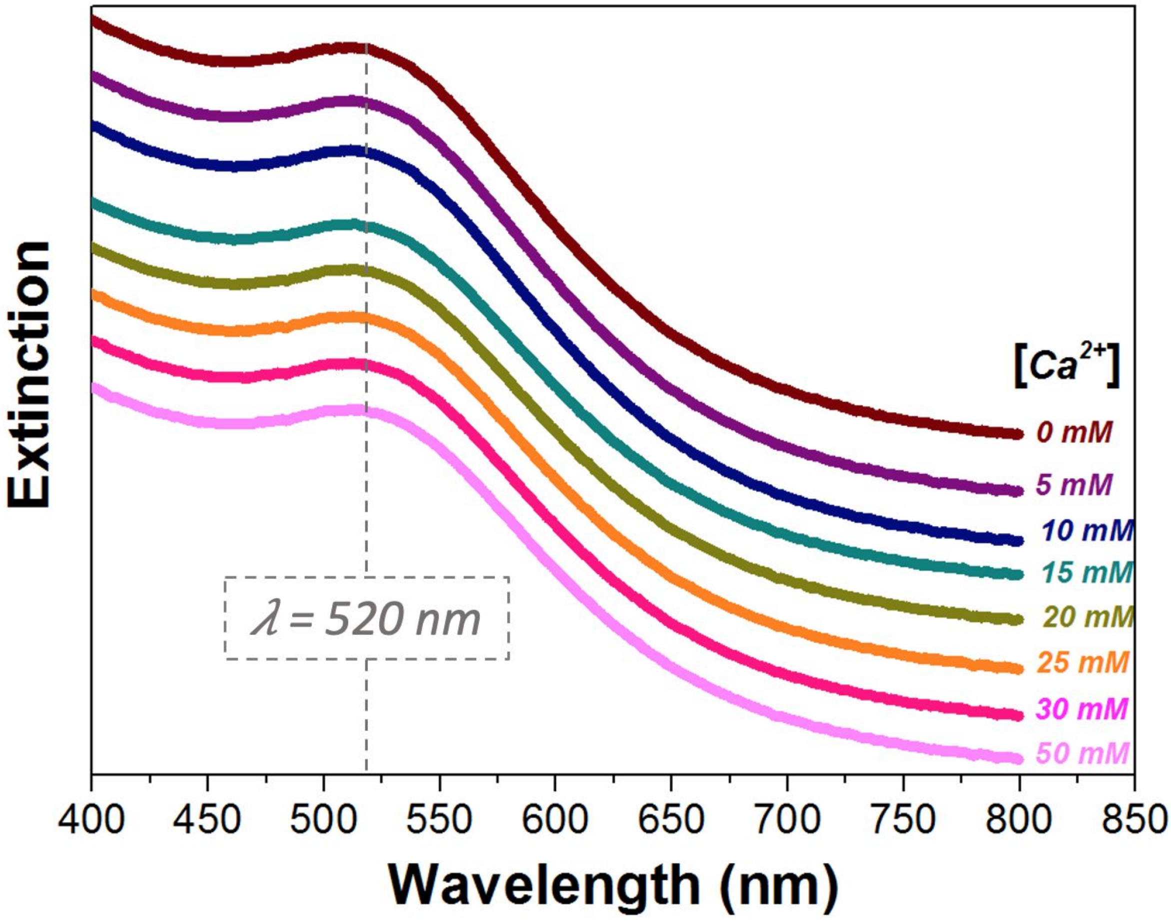

Figure S10. UV-visible extinction spectra of the Au/PEI nanoparticles in aqueous solution containing [Ca^{2+}] = 0 – 50 mM. The suspensions were adjusted to pH = 9 using 100 mM NaOH. No change in the position, shape or intensity of the surface plasmon resonance band at λ = 520 nm is observed when [Ca^{2+}] is increased in the suspensions, indicating that the nanoparticles remain highly stable in solution. The spectra are offset for sake of clarity.

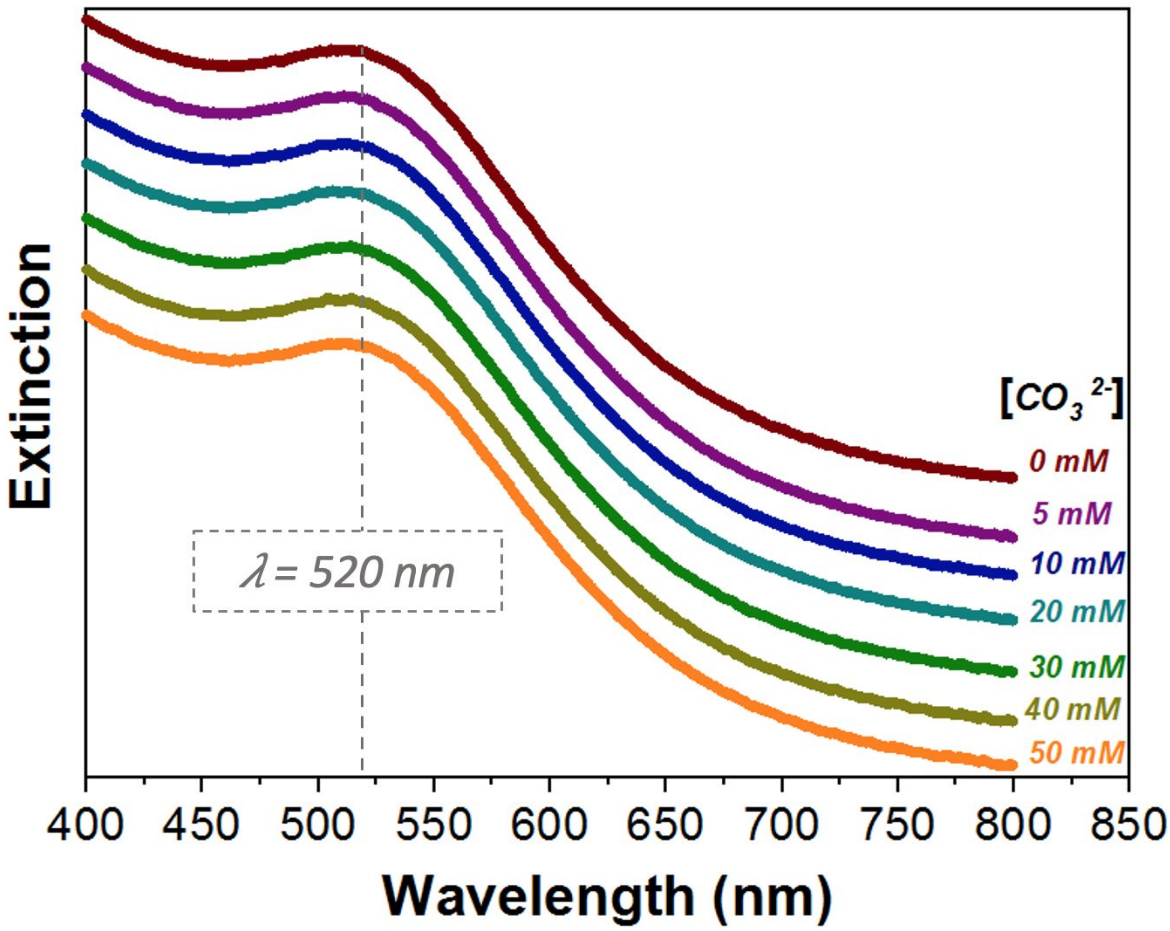

Figure S11. UV-visible extinction spectra of the Au/PEI nanoparticles in aqueous solution containing $[CO_3^{2-}]$ = 0 – 50 mM (using Na_2CO_3). No change in the position, shape or intensity of the surface plasmon resonance band at λ = 520 nm is observed when $[CO_3^{2-}]$ is increased in the suspensions, indicating that the nanoparticles remain highly stable in solution. The spectra are offset for sake of clarity.

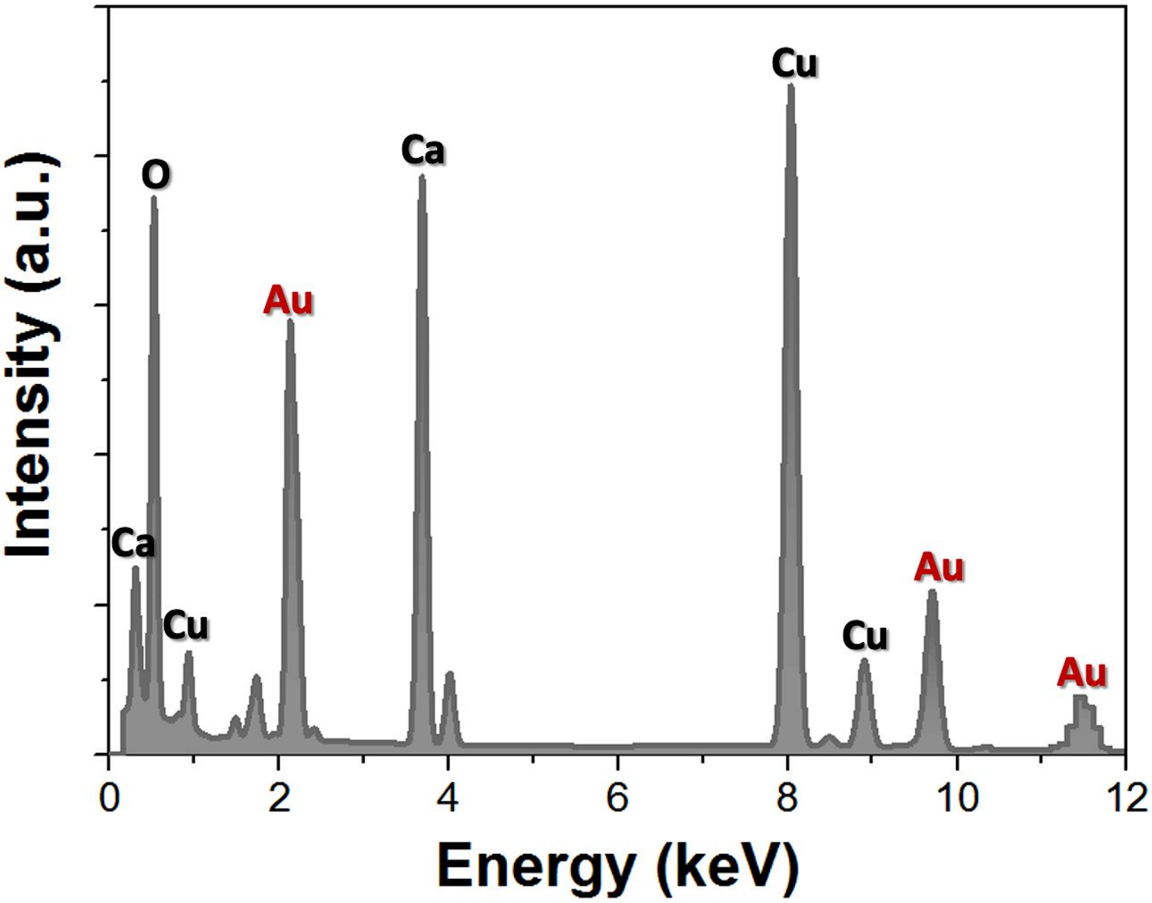

Figure S12. Energy dispersive X-ray (EDX) analysis of Au/PEI/calcite composite crystals showing the characteristic peaks of the gold nanoparticles (Au in red) and the Ca and O from the calcite ($CaCO_3$) host crystals. The copper (Cu) arises from the TEM grid supporting the composite crystals.

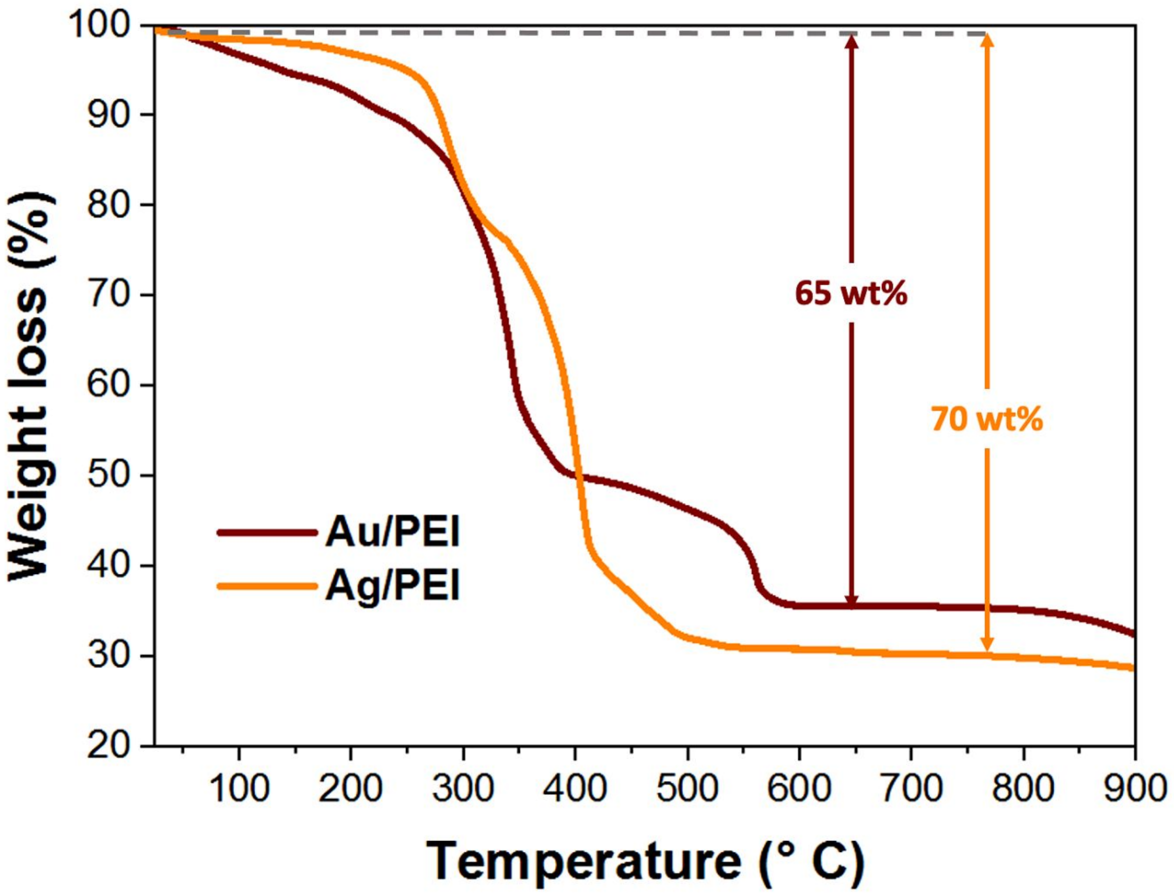

Figure S13. TGA recorded in air of the Au/PEI nanoparticles (red) and Ag/PEI nanoclusters (orange). The weight losses measured between room temperature and ≈ 650°C correspond to the amount of PEI adsorbed on the surface of the metal nanoparticles. The remaining masses correspond to the mass fractions of the Au nanoparticles (35 wt%) and Ag nanoclusters (30 wt%).

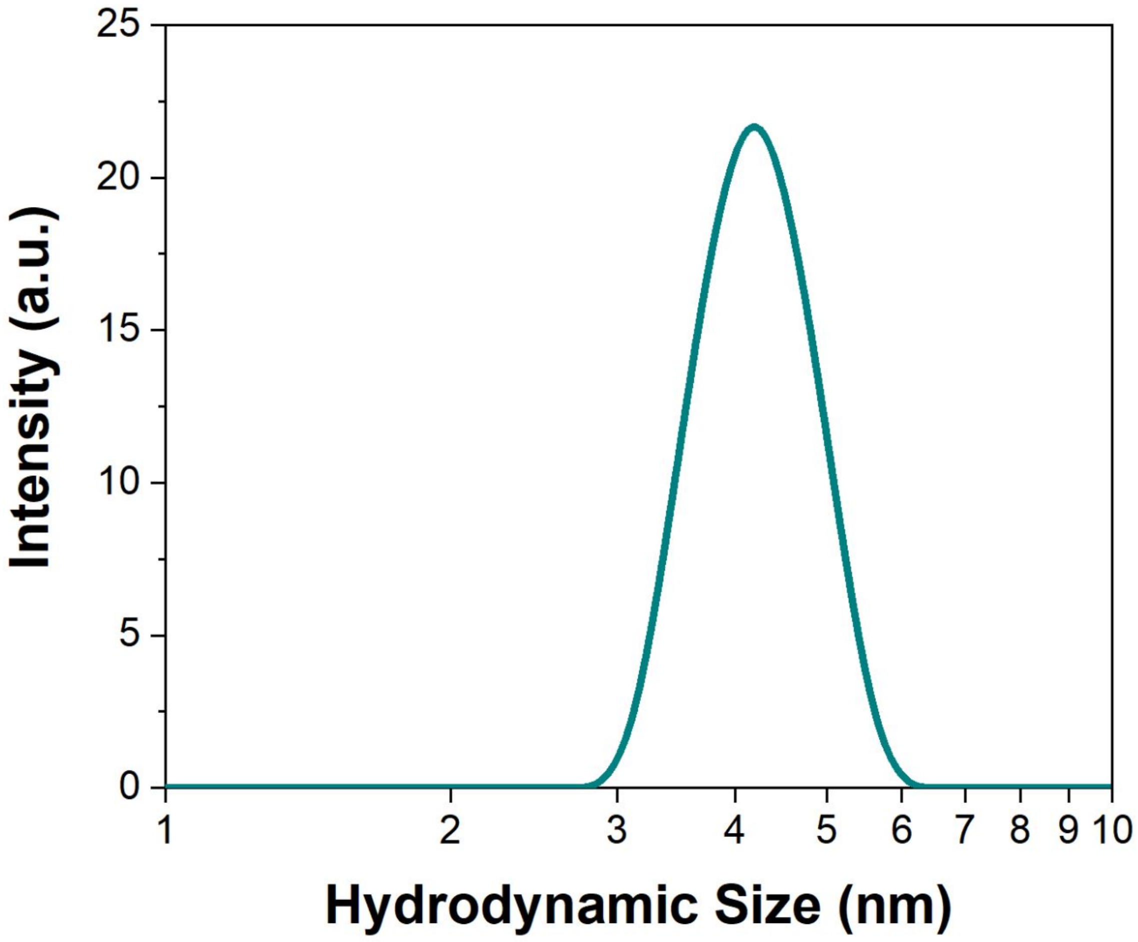

Figure S14. Size distribution analysis of an aqueous solution of the silver nanoclusters surface-functionalized with poly(ethylene imine) (Ag/PEI) (0.10 wt%) obtained by dynamic light scattering (DLS). The nanoparticles have unimodal sizes of ≈ 4 nm.

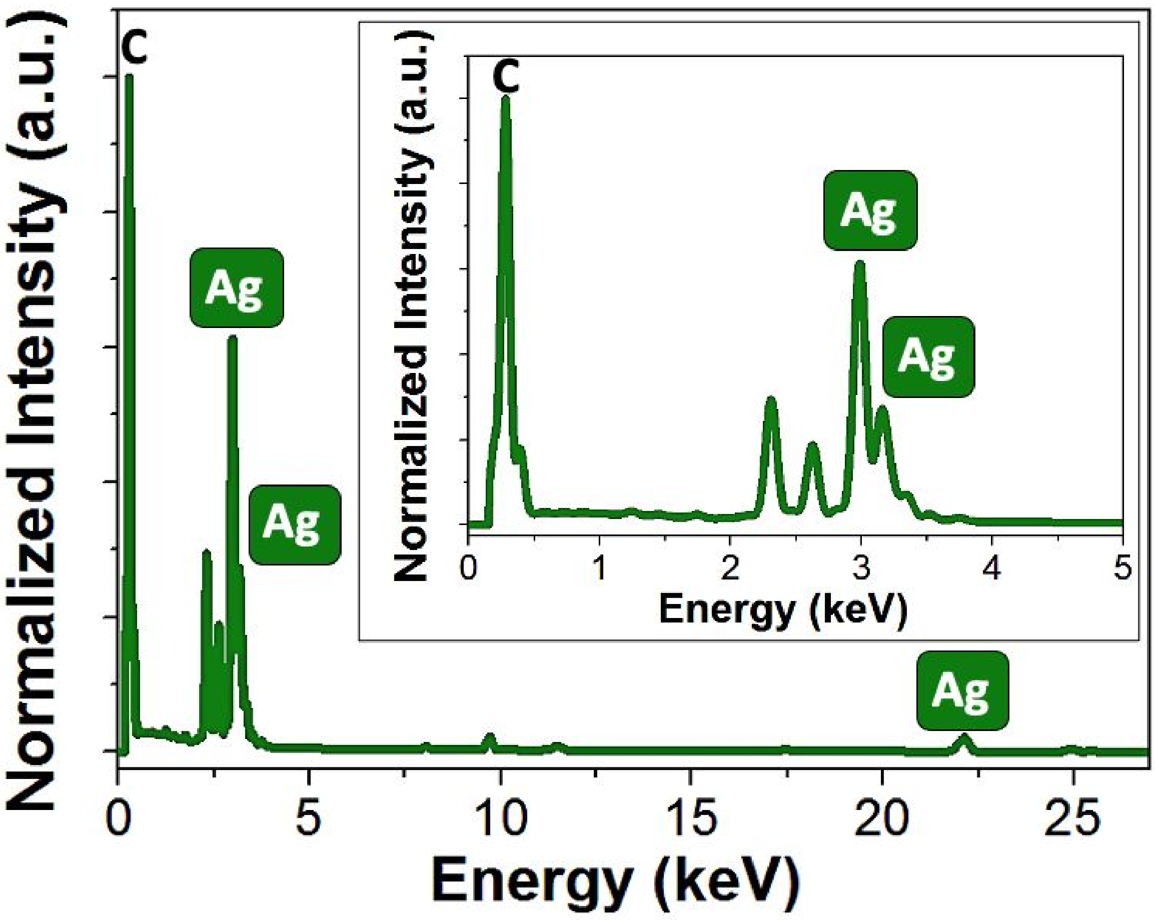

Figure S15. Energy dispersive X-ray (EDX) analysis of Ag/PEI nanoclusters showing the characteristic peaks of silver (Ag in green) and carbon (C) from the carbon film coating the TEM grid.

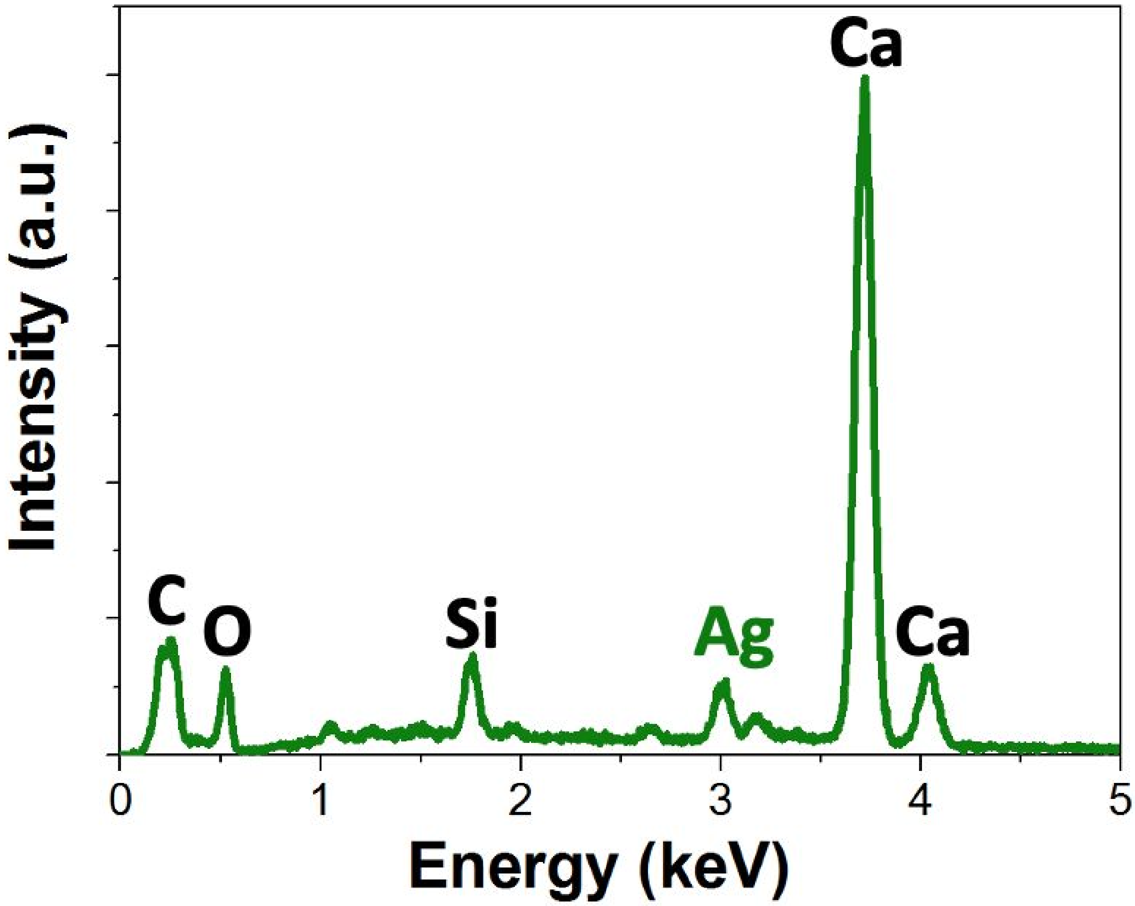

Figure S16. SEM-EDX analysis of Ag/PEI/calcite nanocomposite crystals showing the characteristic peaks of silver (Ag in green) and the Ca, C and O from the calcite host crystal. The silicon peak (Si) corresponds to the silicon wafer supporting the hybrid crystals.

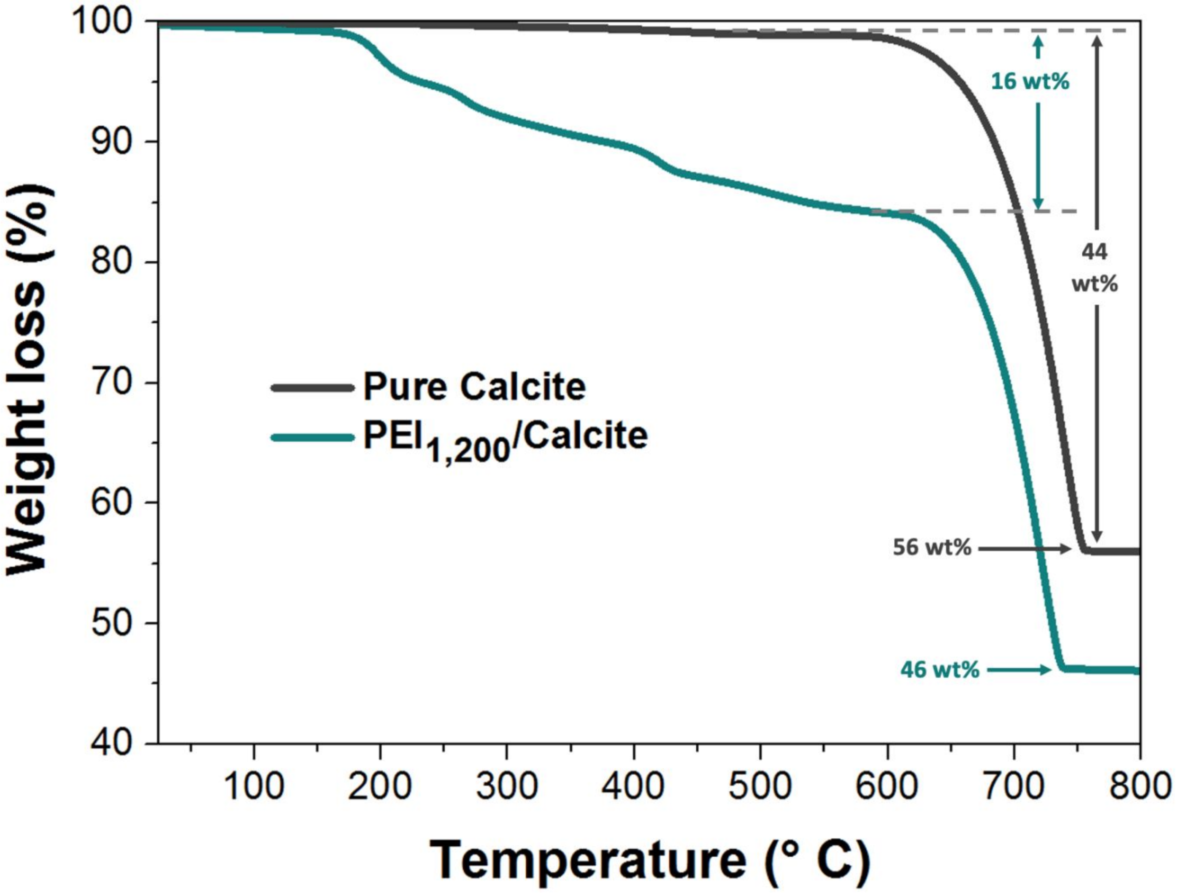

Figure S17. TGA recorded in air for pure calcite (dark grey) and calcite incorporating PEI (M_W = 1,200 g mol^{-1}, cyan). The weight loss of the composites measured between room temperature and ≈ 650°C correspond to the amount of PEI incorporated within calcite.

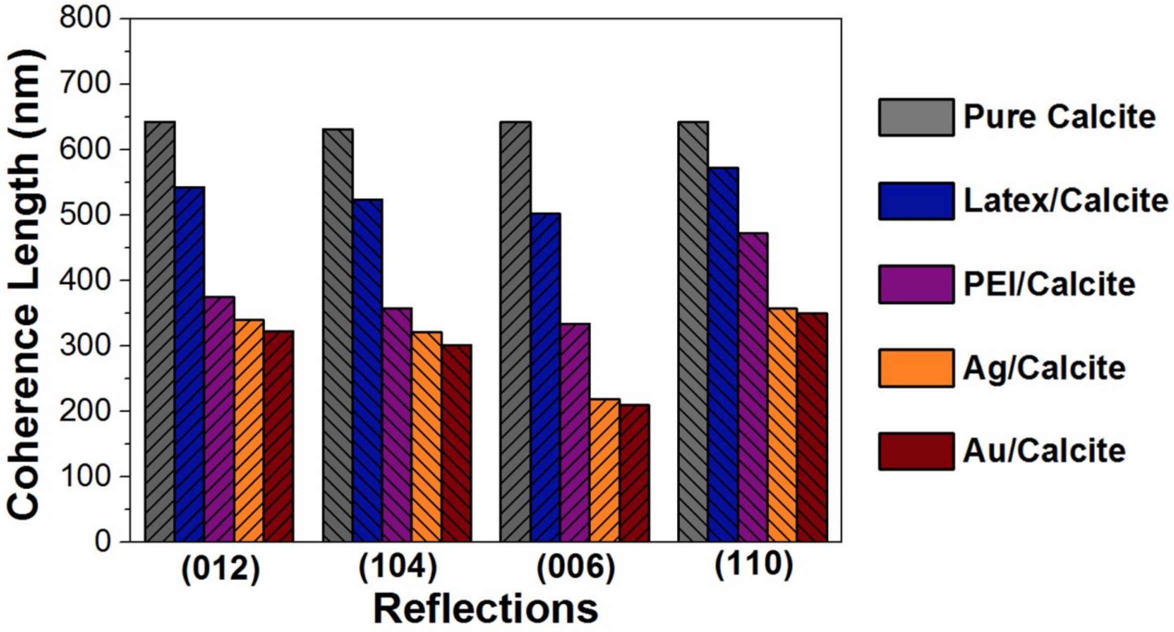

Figure S18. Graph showing the coherence lengths of pure calcite (grey) and calcite incorporating PMMA/PEI nanoparticles (Latex/Calcite, blue), $PEI_{1,200}$ (PEI/Calcite, purple), Ag nanoclusters functionalized with PEI (Ag/Calcite, orange) and Au nanoparticles functionalized with PEI (Au/Calcite, red). The decrease of the coherence lengths of the composite crystals compared to pure calcite indicates the efficient incorporation of the basic additives within the lattice of calcite.[19, 20]

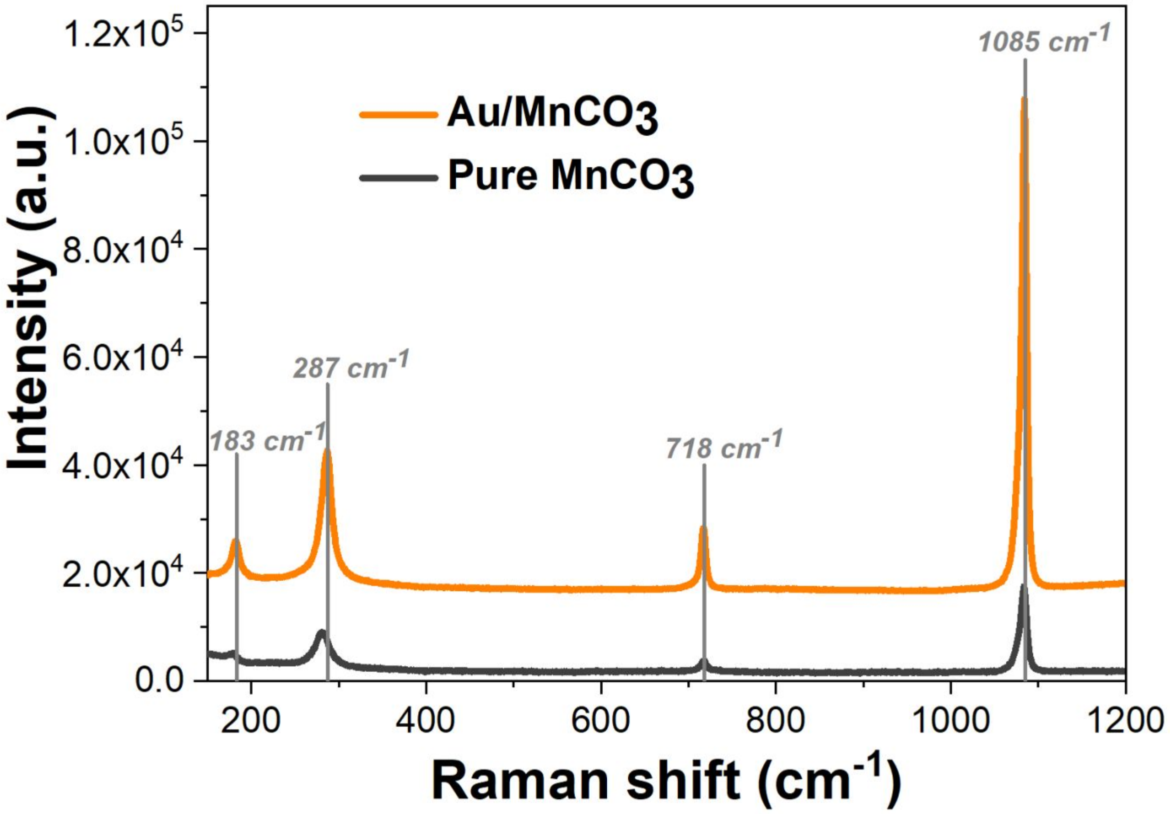

Figure S19. Raman spectra of pure $MnCO_3$ (black) and $MnCO_3$ incorporating Au/PEI nanoparticles (Au/$MnCO_3$, orange) showing the characteristic peaks ν_1 (1085 cm^{-1}), ν_4 (718 cm^{-1}) and lattice modes (183 cm^{-1} and 287 cm^{-1}) of rhodochrosite. Comparison of the Raman spectra intensities shows a surface-enhanced Raman scattering (SERS) effect for the composites when irradiated with a laser source (λ = 785 nm), using 10 sec exposure time and 5% laser power. The spectra are offset for the sake of clarity.

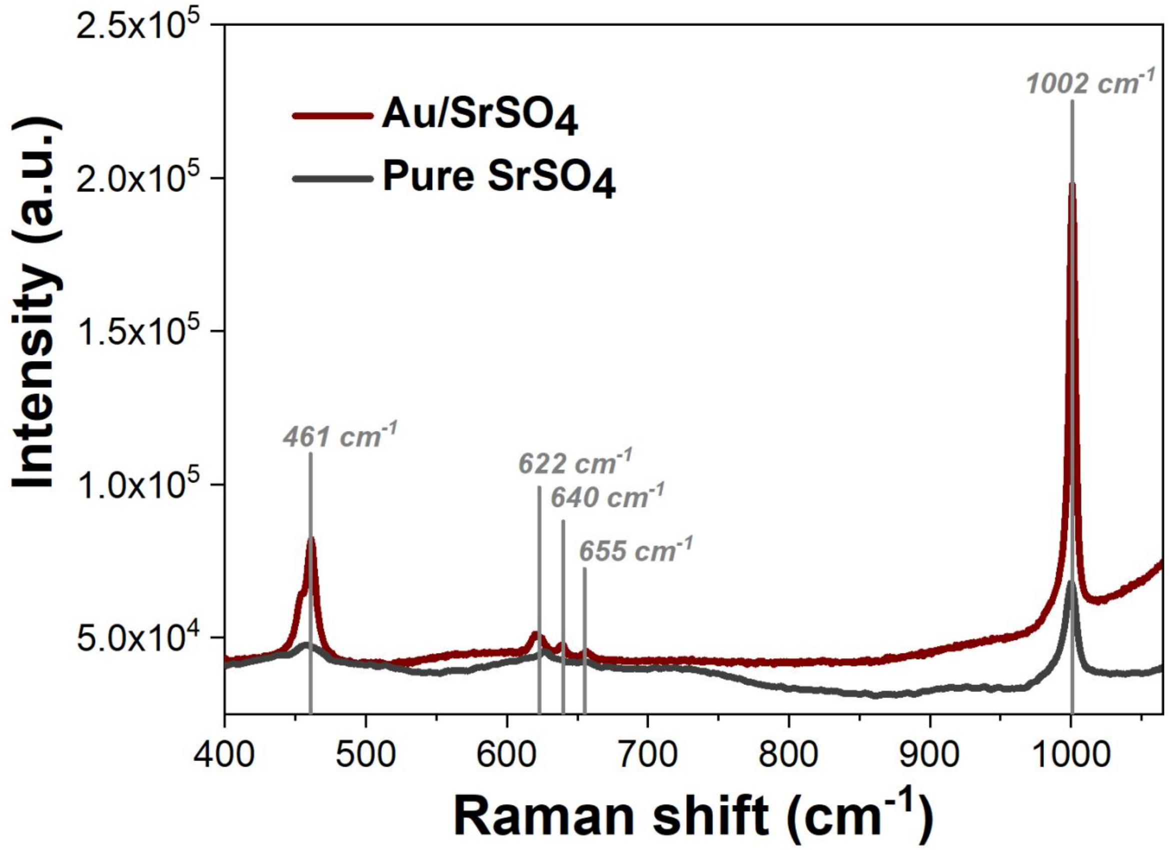

Figure S20. Raman spectra of pure $SrSO_4$ (dark grey) and $SrSO_4$ crystals incorporating Au/PEI nanoparticles (Au/$SrSO_4$, red) showing the characteristic peaks ν_1 (1002 cm^{-1}), ν_4 (622 cm^{-1}, 640 cm^{-1} and 655 cm^{-1}) and ν_2 (461 cm^{-1}) of celestine. Comparison of the Raman spectra intensities shows a surface-enhanced Raman scattering (SERS) effect for the composites when irradiated with a laser source (λ = 785 nm), using 10 sec exposure time and 5% laser power.

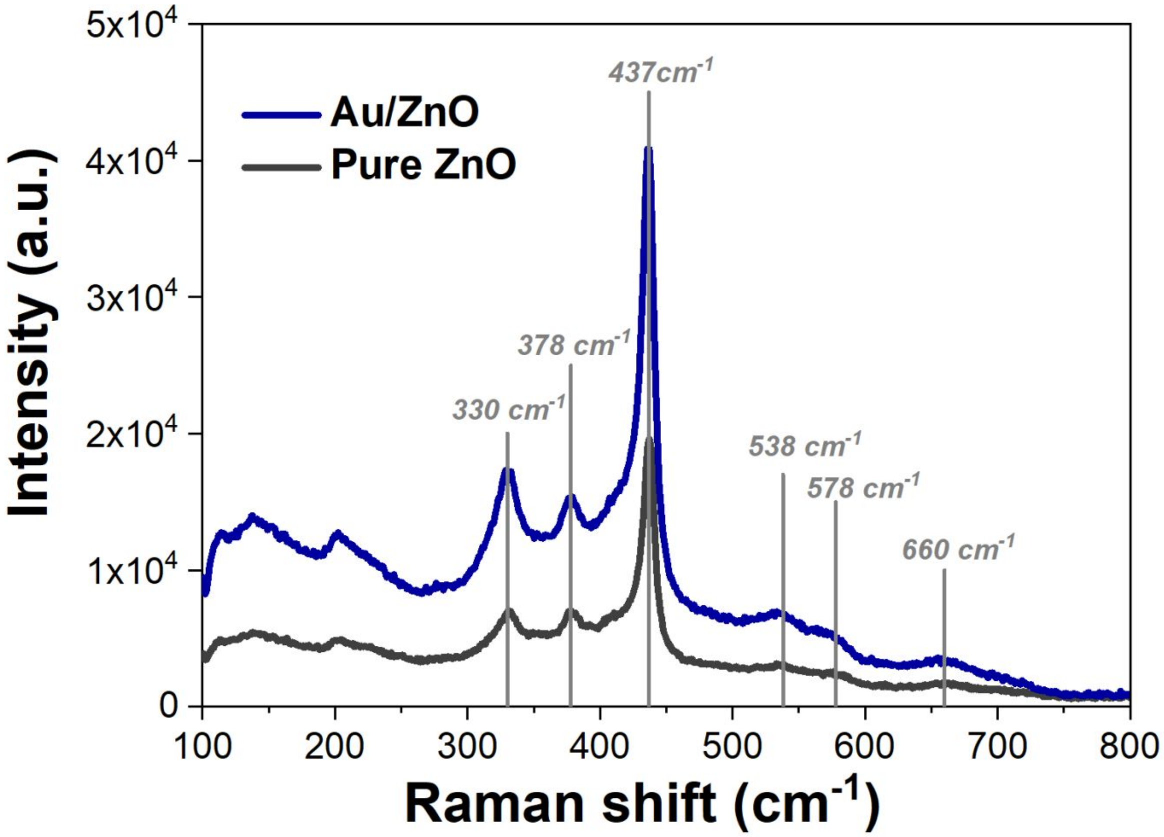

Figure S21. Raman spectra of pure ZnO (dark grey) and ZnO crystals incorporating Au/PEI nanoparticles (Au/ZnO, blue) showing the characteristic peaks 330 cm^{-1}, 378 cm^{-1}, 437 cm^{-1} and 538 cm^{-1}, 578 cm^{-1} and 660 cm^{-1} of zincite. Comparison of the Raman spectra intensities shows a surface-enhanced Raman scattering (SERS) effect for the composites when irradiated with a laser source (λ = 785 nm), using 10 sec exposure time and 5% laser power.

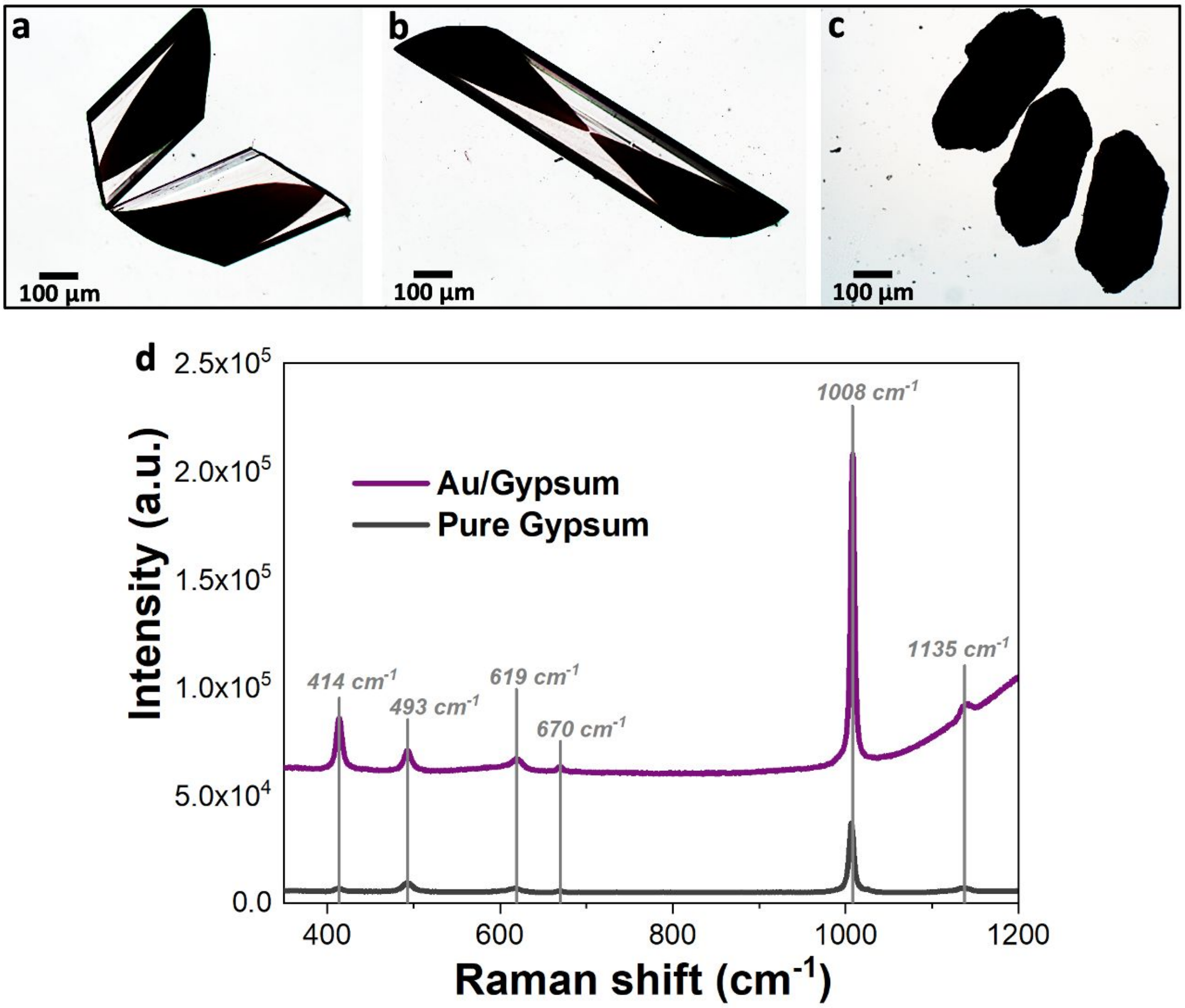

Figure S22. Optical images showing the zoning effect induced by the incorporation of Au/PEI (0.1 wt%) within gypsum ($CaSO_4 \cdot 2H_2O$) (a-b), and Au/PEI incorporated throughout the whole structure of gypsum when a higher [Au/PEI] = 0.25 wt% is introduced in the mineralization solution (c). (d) Raman spectra of pure $CaSO_4$ (dark grey) and $CaSO_4$ crystals incorporating Au/PEI nanoparticles (Au/Gypsum, purple) showing the characteristic peaks ν_4 (SO_4^{2-}) (1135 cm^{-1}), ν_3 (SO_4^{2-}) (1008 cm^{-1}), ν_2 (H_2O) (619 cm^{-1} and 670 cm^{-1}), ν_1 (H_2O) (493 cm^{-1}) and ν_3 (H_2O) (414 cm^{-1}) of gypsum. Comparison of the Raman spectra intensities shows a surface-enhanced Raman scattering (SERS) effect for the composites when irradiated with a laser source (λ = 785 nm), using 10 sec exposure time and 5% laser power. The spectra are offset for the sake of clarity.

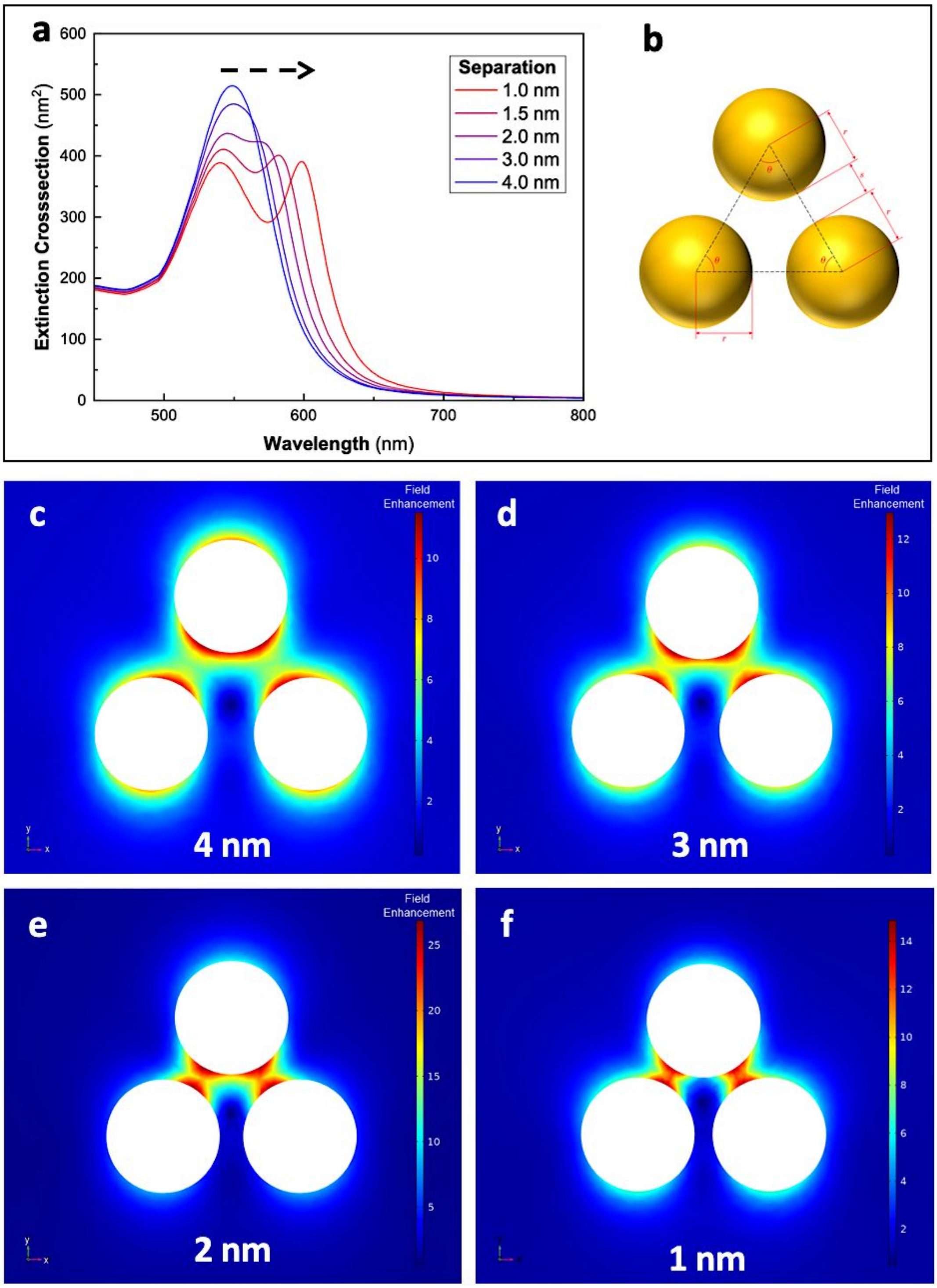

Figure S23. (a) Simulated extinction spectra for 5 nm AuNPs trimers incorporated in calcite with varying interparticle separations between 0.5 nm and 4 nm. (b) Diagram showing the geometry of the simulation for the trimer configuration. (c-f) FEM simulated field enhancement $|E|/|E_0|$ along the y-axis for the 5 nm AuNPs trimers with an interparticle gap of (c) 4 nm, (d) 3 nm, (e) 2 nm, (f) and 1 nm.

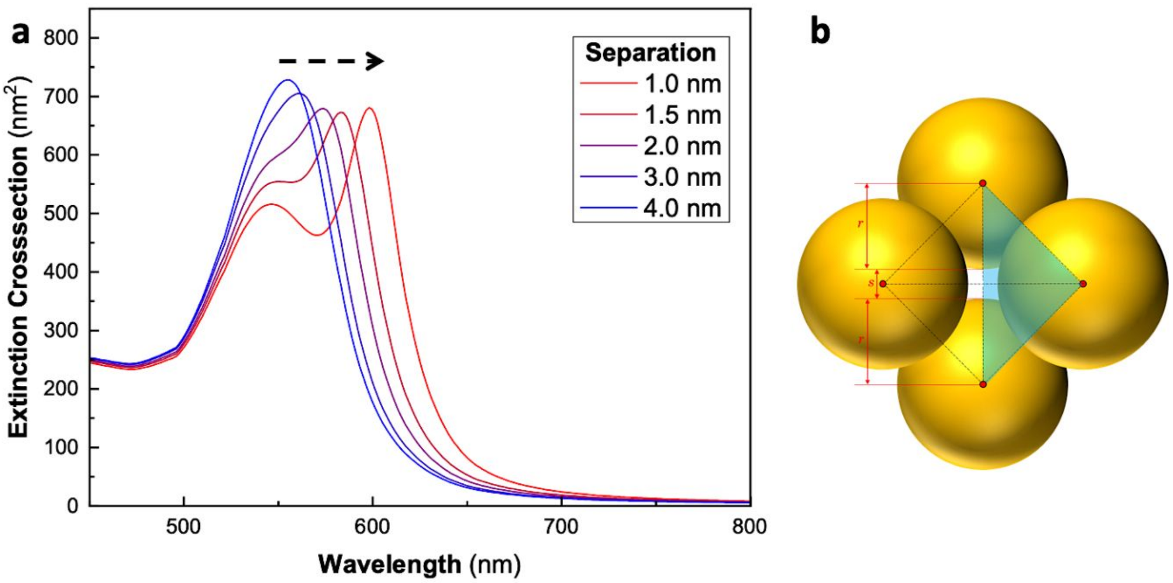

Figure S24. (a) Simulated extinction spectra for 5 nm AuNPs tetramers incorporated in calcite with an interparticle separation between 0.5 nm and 4 nm. (b) Diagram showing the geometry of the simulation for the trimer configuration.

REFERENCES

1. Kook, J.-W.; Lee, J.; Hwang, K.; Park, I.; Kim, J., Synthesis and Characterization of Poly (Methyl Methacrylate)/Polyethylenimine Grafting Core-Shell Nanoparticles for CO2 Adsorption Using Soap-Free Emulsion Copolymerization. *AMPC* **2016,** 6, (7), 220-229.

2. Meng, L.; Zhu, Q.; Yin, J.-H.; Xu, N., Polyethyleneimine protected silver nanoclusters luminescence probe for sensitive detection of cobalt (II) in living cells. *J. Photochem. Photobiol. B* **2017,** 173, 508-513.

3. Ihli, J.; Bots, P.; Kulak, A.; Benning, L. G.; Meldrum, F. C., Elucidating Mechanisms of Diffusion-Based Calcium Carbonate Synthesis Leads to Controlled Mesocrystal Formation. *Adv. Funct. Mater.* **2013,** 23, (15), 1965-1973.

4. Johnson, P. B.; Christy, R. W., Optical Constants of the Noble Metals. *Phys. Rev. B Condens. Matter* **1972,** 6, (12), 4370-4379.

5. Ghosh, G., Dispersion-equation coefficients for the refractive index and birefringence of calcite and quartz crystals. *Opt. Commun.* **1999,** 163, (1), 95-102.

6. Plimpton, S., Fast Parallel Algorithms for Short-Range Molecular Dynamics. *J. Comput. Phys.* **1995,** 117, (1), 1-19.

7. Raiteri, P.; Demichelis, R.; Gale, J. D., Thermodynamically Consistent Force Field for Molecular Dynamics Simulations of Alkaline-Earth Carbonates and Their Aqueous Speciation. *J. Phys. Chem. C* **2015,** 119, (43), 24447-24458.

8. Wu, Y.; Tepper, H. L.; Voth, G. A., Flexible simple point-charge water model with improved liquid-state properties. *J. Chem. Phys.* **2006,** 124, (2), 024503.

9. Wang, J.; Wang, W.; Kollman, P. A.; Case, D. A., Automatic atom type and bond type perception in molecular mechanical calculations. *J. Mol. Graph. Model* **2006,** 25, (2), 247-260.

10. Wang, J.; Wolf, R. M.; Caldwell, J. W.; Kollman, P. A.; Case, D. A., Development and testing of a general amber force field. *J. Comput. Chem.* **2004,** 25, (9), 1157-74.

11. Darkins, R. Computational insight into the molecular mechanisms that control the growth of inorganic crystals. University College London, 2017.

12. Tribello, G. A.; Bonomi, M.; Branduardi, D.; Camilloni, C.; Bussi, G., PLUMED 2: New feathers for an old bird. *Comput. Phys. Commun.* **2014,** 185, (2), 604-613.

13. Raiteri, P.; Laio, A.; Gervasio, F. L.; Micheletti, C.; Parrinello, M., Efficient Reconstruction of Complex Free Energy Landscapes by Multiple Walkers Metadynamics. *J. Phys. Chem. B* **2006,** 110, (8), 3533-3539.

14. Barducci, A.; Bussi, G.; Parrinello, M., Well-Tempered Metadynamics: A Smoothly Converging and Tunable Free-Energy Method. *Phys. Rev. Lett.* **2008,** 100, (2), 020603.

15. Penkman, K. E.; Kaufman, D. S.; Maddy, D.; Collins, M. J., Closed-system behaviour of the intra-crystalline fraction of amino acids in mollusc shells. *Quat. Geochronol.* **2008,** 3, (1-2), 2-25.

16. Dolomanov, O. V.; Bourhis, L. J.; Gildea, R. J.; Howard, J. A.; Puschmann, H., OLEX2: a complete structure solution, refinement and analysis program. *J. Appl. Crystallogr.* **2009,** 42, (2), 339-341.

17. Sheldrick, G. M., SHELXT–Integrated space-group and crystal-structure determination. *Acta Crystallogr. A* **2015,** 71, (1), 3-8.

18. Sheldrick, G. M., Crystal structure refinement with SHELXL. *Acta Cryst. C* **2015,** 71, (1), 3-8.

19. Kim, Y.-Y.; Carloni, J. D.; Demarchi, B.; Sparks, D.; Reid, D. G.; Kunitake, Miki E.; Tang, C. C.; Duer, M. J.; Freeman, C. L.; Pokroy, B.; Penkman, K.; Harding, J. H.; Estroff, L. A.; Baker, S. P.; Meldrum, F. C., Tuning hardness in calcite by incorporation of amino acids. *Nat. Mater.* **2016,** 15, (8), 903-910.

20. Kim, Y.-Y.; Semsarilar, M.; Carloni, J. D.; Cho, K. R.; Kulak, A. N.; Polishchuk, I.; Hendley IV, C. T.; Smeets, P. J. M.; Fielding, L. A.; Pokroy, B.; Tang, C. C.; Estroff, L. A.; Baker, S. P.; Armes, S. P.; Meldrum, F. C., Structure and Properties of Nanocomposites Formed by the Occlusion of Block Copolymer Worms and Vesicles Within Calcite Crystals. *Adv. Funct. Mater.* **2016,** 26, (9), 1382-1392.
